# Enhanced Inhibition of *Trametes versicolor* by Structurally Modified Medicarpin: In Vitro Evaluation and In Silico Insights into Laccase Binding

**DOI:** 10.3390/ijms27062878

**Published:** 2026-03-22

**Authors:** Santiago José Guevara-Martínez, José Domingo Rivera-Ramírez, Rebeca Escutia-Gutiérrez, Marco Antonio Pérez-Cisneros, Francisco Villanueva-Mejía, Stephanie García-Zavala, Rafael Herrera-Bucio, Fredy Geovannini Morales-Palacios

**Affiliations:** 1 Laboratorio de Química Farmacéutica, Departamento de Farmacobiología, Centro Universitario de Ciencias Exactas e Ingenierías, Universidad de Guadalajara, Boulevard Gral. Marcelino García Barragán 1421, Olímpica, Guadalajara 44430, Jalisco, Mexico; santiago.guevara@academicos.udg.mx (S.J.G.-M.); domingo.rivera@academicos.udg.mx (J.D.R.-R.); 2 Departamento de Biología Molecular y Genómica, Centro Universitario de Ciencias de la Salud, Universidad de Guadalajara, Sierra Mojada 950, Independencia Oriente, Guadalajara 44430, Jalisco, Mexico; rebeca.escutia@academicos.udg.mx; 3 Departamento de Electrofotónica, Centro Universitario de Ciencias Exactas e Ingenierías, Universidad de Guadalajara, Boulevard Gral. Marcelino García Barragán 1421, Olímpica, Guadalajara 44430, Jalisco, Mexico; marco.perez@cucei.udg.mx; 4 Laboratorio de Herramientas Computacionales, Instituto Tecnológico de Pabellón de Arteaga, Carretera a la Estación de Rincón de Romos, Km 1, Aguascalientes 20267, Aguascalientes, Mexico; francisco.vm@pabellon.tecnm.mx; 5 Instituto de Investigación Químico Biológicas, Universidad Michoacana de San Nicolás de Hidalgo, Francisco J. Múgica, s/n, Morelia 58030, Michoacán, Mexico; 1106886k@umich.mx

**Keywords:** natural products, wood preservatives, *Trametes versicolor*, laccase, docking, dynamics

## Abstract

Medicarpin, a natural pterocarpan phytoalexin, contributes to tree defense against microbial decay, particularly from the aggressive white-rot fungus *Trametes versicolor*, an ASTM standard for wood durability testing. To improve upon the inhibitory effect of medicarpin against this fungus (150 mg/L), eleven derivatives were synthesized and evaluated. The acetylated analog demonstrated superior activity, achieving complete growth inhibition at 100 mg/L. To establish a structure–activity relationship, molecular docking was performed on the copper cluster on fungal laccase, the primary oxidative enzyme of *T. versicolor*. The acetylated derivative bound the T1 copper site with a more favorable free energy (−8.5 kcal/mol) than the parent compound, exhibiting enhanced stabilizing interactions and a binding pose anchored closer to the trinuclear copper cluster (TNC). These results were corroborated by 80 ns molecular dynamics simulations, confirming complex stability and the persistence of key interactions. This study demonstrates that targeted chemical modification of natural phytoalexins can significantly improve their antifungal potency. The superior performance of the acetylated medicarpin derivative, linked to optimized binding at the laccase active site, establishes a clear structure–activity relationship and highlights the potential of such engineered compounds as leads for next-generation, bio-inspired wood preservatives.

## 1. Introduction

Medicarpin is a natural isoflavonoid belonging to the pterocarpan subclass, found in a wide range of leguminous plants and trees. Its sources include *Medicago sativa* (alfalfa), *Lathyrus sativus*, species *Dalbergia*, *Sophora japonica*, *Medicago truncatula*, *Trigonella foenum-graecum*, *Pterocarpus santalinus*, *Canavalia lineata*, and *Andira inermis* [1,2,3,4,5,6,7]. This broad distribution, particularly among species with traditional medicinal uses, has prompted extensive investigation into this biological potential.

Studies have reported that medicarpin exhibits a diverse array of pharmacological activities. These include antibacterial effects [8], neuroprotective properties relevant to memory impairment and Parkinson’s disease [7,9], and phytoestrogenic activity that promotes bone formation and prevents osteopenia [10,11,12], anti-arthritic potential [13], and pro-apoptotic effects against leukemia cells [14,15]. While these pharmacological properties are well documented, they do not fully capture the ecological and molecular significance of medicarpin in plant defense. Of particular relevance to material science and wood preservations is its native roles as a phytoalexin, a compound synthesized by plants in response to pathogen attack, which confers natural resistance against wood-decaying fungi.

From a molecular and materials science perspective, medicarpin is particularly relevant due to its inherent role as a phytoalexin, biosynthesized by plants in response to biotic and abiotic stress. Its accumulation is induced by mechanical injury, temperature fluctuations, culture medium modifications, or pathogen infection [16,17,18,19]. This defensive function reflects a natural propensity for antimicrobial activity, including antifungal action against wood-decaying organisms. In this context, medicarpin represents a promising natural lead compound for the development of environmentally benign, bio-inspired wood preservatives.

A representative example is the tree *Andira inermis* (W. Wright) DC, which exhibits remarkable natural durability against environmental and biological degradation. This resistance has been largely attributed to the high content of extractives in its heartwood and bark, among which medicarpin is a prominent constituent [20].

Consistent with this hypothesis, our research group has previously demonstrated the antifungal activity of medicarpin against the white-rot fungus *Trametes versicolor*, inhibiting fungal growth at a concentration of 150 mg/mL [21]. *T. versicolor* is one of the most aggressive and widely distributed wood-decaying fungi, capable of degrading cellulose, hemicellulose, and lignin and utilizing them as carbon sources to support its own development and growth [22,23,24]. Due to its high degradative capacity, American Society for Testing and Materials (ASTM) standards recommend its use in accelerated laboratory assays to evaluate wood durability, a property closely linked to the presence of secondary metabolites [25,26,27].

The wood-degrading capacity of *T. versicolor* is primarily mediated by ligninolytic enzymes involved in oxidative processes, including lignin peroxidase (LiP), manganese peroxidase (MnP), and laccase [28,29,30]. However, numerous studies have demonstrated that laccase production by the fungus *T. versicolor* is typically higher compared to LiP and MnP enzymes. Moreover, the expression of these enzymes is dependent on growth medium conditions (substrate, temperature, culture medium, etc.), and in some particular cases, LiP is not even secreted. In contrast, laccase exhibits a broader substrate oxidation spectrum (compounds), whereas LiP and MnP show oxidative activity toward more specific substrates [31,32,33]. For these reasons, most fungal studies in the literature have focused on the enzymatic (redox) processes of laccase [34,35].

Laccase (benzediol: oxygen reductase), a multicopper oxidase containing one type 1 (T1) copper site and a trinuclear cluster (TNC) composed of one type 2 (T2), and two type 3 (T3) copper atoms. Substrate oxidation occurs at the T1 site, followed by electron transfer to the TNC, where molecular oxygen is reduced to water [36,37,38]. This catalytic system enables laccase to oxidize a broad range of phenolic and non-phenolic substrates, aromatic amines and amides, benzothiols, hydroxyindoles, biphenyls, and pentachlorophenols, creating oxidative conditions that support fungal growth and wood degradation.

Although medicarpin has demonstrated inhibitory effects against *T. versicolor* and activity toward laccase, its antifungal potential remains amenable to optimization. Despite the demonstrated antifungal activity of medicarpin against *T. versicolor*, the molecular determinants governing its interaction with fungal laccase, as well as the extent to which rational structural modification can enhance binding affinity and inhibitory efficiency at the catalytic copper centers, remain insufficiently understood. In this study, the phenolic hydroxyl group of medicarpin was selected as a strategic site for structural modification. Functionalization at this position offers a rational approach to enhance antifungal efficacy while modulating physicochemical and molecular interaction properties. This strategy is supported by numerous studies on natural products, where targeted derivatization has led to improved biological activity and molecular performance [39,40,41]. Accordingly, the present work aims to advance the development of antifungal agents derived from natural products, with potential applications in wood preservation.

## 2. Results

### 2.1. Isolation and Structural Modification of Medicarpin

Medicarpin (**1**) was isolated from the ethyl acetate-soluble heartwood extract of *Andira inermis*. Its identity and purity were confirmed by comparing its ^1^H and ^13^C NMR spectral data with literature values [21,42].

Subsequently, the phenolic hydroxyl group of compound **1** was functionalized to generate a library of eleven derivatives (**2**–**12**). The synthetic modifications included O-alkylation (compounds **2**, **3**, **4**), acetylation (**5**), acylation with various aliphatic and aromatic acids (**6**, **7**, **8**, **10**, **11**, **12**), and carbamate formation (**9**). The complete series is presented in Table 1.

All derivatives retained the characteristic NMR features of the pterocarpan scaffold, while substitution at the hydroxyl position was confirmed by diagnostic spectral changes. Ether derivatives (**2**–**4**) lacked ester carbonyl signals in the ^13^C NMR spectra and displayed alkyl or benzylic resonances consistent with O-alkylation. In contrast, ester and carbamate derivatives (**5**–**12**) exhibited new carbonyl resonances in the δ 153–176 ppm range, with side-chain-dependent signals confirming successful functionalization. Detailed spectroscopic data are provided in the Experimental Section and NMR spectra are available in the Supporting Information; the key diagnostic resonances are summarized in Table 2.

It should be noted that the NMR spectra of derivatives **2**–**4** and **6**–**8** exhibit additional signals beyond those corresponding to each individual derivative, but with similar chemical shifts across all of them (δ-^1^H ~5.9 ppm; δ-^13^C ~148, 142, and 96 ppm). These derivatives share the common feature of having flexible alkyl substituents. In contrast, derivatives **1**, **5**, and **9**–**12**, which differ by having substituents that restrict conformational states, do not show these additional signals in their NMR spectra (Figures S1–S37).

This signal duplication observed for derivatives **2**–**4** and **6**–**8** is attributed to the presence of two slowly interconverting conformations (rotamers) resulting from restricted rotation around the Ar–O–R or Ar–O–C(O)R bonds in compounds bearing flexible alkyl chains. As this interconversion is slow on the NMR timescale, two sets of signals—typically in a 1:1 ratio—are detected in both ^1^H and ^13^C spectra.

It is important to note that the additional signals correspond to the same molecular connectivity of the derivatives and are not due to structural inconsistencies or impurities, but rather to conformational behavior. This can be observed in the two-dimensional spectra (DEPT and TOCSY) of the indicated derivatives, available in the Supporting Information, where it is demonstrated that these signals are not associated with any hybridization artifacts in the DEPT experiment or with any type of correlation.

Furthermore, the identity and purity of (+)-medicarpin **1** were further verified by HPLC analysis (Figure S3), which revealed a single, well-resolved peak with retention time and UV absorbance consistent with literature reports [43], supporting its use as a reference compound in biological assays.

### 2.2. Antifungal Activity Against Trametes versicolor

The antifungal activity of medicarpin (**1**) and derivatives **2**–**12** was evaluated against *T. versicolor* using an agar incorporation assay (diffusion method) at three concentrations. Consistent with previous reports, compound **1** was included as an internal control and completely inhibited fungal growth at 150 mg/L and maintained 80% inhibition at 100 mg/L [21].

**Figure 1:**
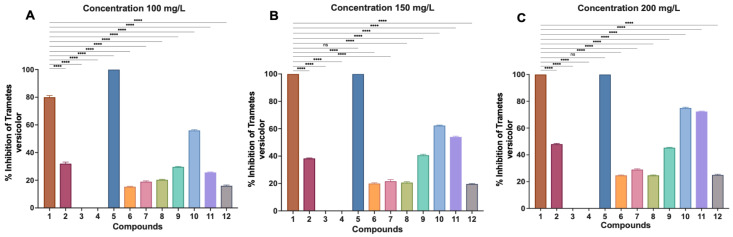
Antifungal activity of medicarpin (**1**) and its derivatives (**2**–**12**) against *Trametes versicolor.* Inhibition percentages were evaluated at three concentrations: (**A**) 100 mg/L, (**B**) 150 mg/L, and (**C**) 200 mg/L. Bars represent the mean inhibition percentage ± standard deviation (SD) of three independent experiments. Statistical significance relative to the control ins indicated as **** *p* < 0.0001; ns = not significant.

At 100 mg/L, the control compound (**1**) exhibited strong antifungal activity with approximately 80% inhibition (Figure 1A). Most derivatives showed reduced activity relative to the control. Compound **5** was the only derivative achieving complete inhibition (100%), surpassing the activity of medicarpin at this concentration. Moderate inhibition was observed for compounds **10** (56%) and **2** (32%), whereas compounds **9** and **11** showed lower activity (30% and 26%, respectively). The remaining derivatives (**6**–**8**, **12**) exhibited weak. Inhibition (15–21%), while compounds **3** and **4** were completely inactive.

At 150 mg/L, compound **1** produced complete inhibition (100%) (Figure 1B). Compound **5** maintained complete inhibition, with activity comparable to the control. Several derivatives showed moderate increases in activity, with compounds **10** and **11** reaching 62% and 54% inhibition, respectively, while compound **9** reached 41%. Compounds **2**, **6**–**8**, and **12** displayed low activity (19–38%), and derivatives **3** and **4** remained inactive.

Most structural modifications resulted in diminished antifungal efficacy. Ether derivatives **3** and **4** were inactive across all concentrations, while derivative **2** showed moderate inhibition only at 200 mg/L. Aliphatic ester derivatives (**6**–**8**) and carbamate **9** exhibited weak to moderate activity (15–45%), whereas aromatic esters **10** and **11** displayed improved inhibition at 200 mg/L (>70%), although none matched the potency of the parent compound.

**Figure 2:**
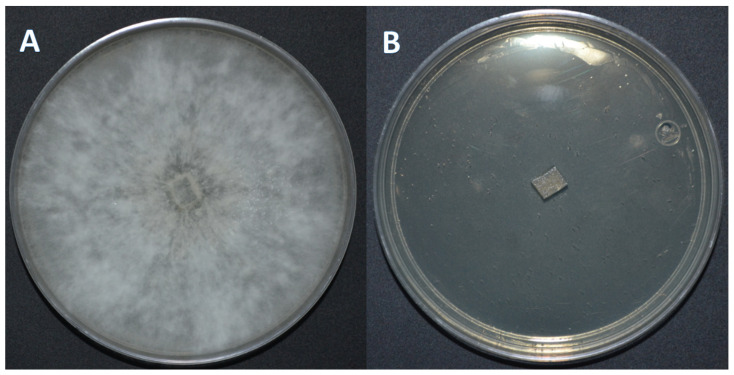
Complete inhibition of *Trametes versicolor* by the acetylated medicarpin derivative **5** at 100 mg/L (**B**), compared to the positive control showing fungal growth (**A**).

Notably, acetylate derivative **5** demonstrated enhanced antifungal potency, achieving complete inhibition at 100 mg/L. This represents an approximate 33% reduction in effective concentration compared to medicarpin (**1**), which required 150 mg/L for the same effect (Figure 1 and Figure 2). At 75 mg/L, derivative **5** exhibited partial 80% inhibition, confirming a concentration-dependent response. The minimum inhibitory concentration for compound **5** therefore lies between 75 and 100 mg/L.

It is worth mentioning that these concentrations are within the inhibitory ranges of various antifungal compounds [44,45], as well as for compounds and extracts of natural origin that the literature has described with good inhibitory activity against *T. versicolor* [46,47,48].

### 2.3. Structure–Activity Relationships

Overall, these results indicate that subtle structural modification of medicarpin can significantly influence antifungal activity. While bulkier ether and aliphatic ester substituents reduced efficacy, acetylation at the phenolic hydroxyl group enhanced activity, suggesting that minimal and strategically positioned modifications can improve biological performance without altering the core pterocarpan scaffold.

## 3. Discussion

While (+)-medicarpin (**1**) has been previously isolated from *Dalbergia congestiflora* Pittier [21], *Andira inermis* heartwood was selected as the source material in this study due to the conservation status of *D. congestiflora*, which is listed as an endangered species [49,50]. Comparative ^1^H/^13^C NMR and HPLC analyses confirmed the compound isolated from *A. inermis* was chemically identical to (+)-medicarpin (**1**), thereby validating its use for subsequent derivatization and biological evaluation.

The design of medicarpin derivatives was guided by two key considerations: preserving the native pterocarpan scaffold, given the limited number of natural products reported to inhibit *T. versicolor*, while selectively modifying the phenolic hydroxyl group through simple and reproducible chemical transformations. Etherification, acetylation, and acylation were therefore selected, as these reactions represent classical hydroxyl functionalization strategies readily accessible under standard laboratory conditions [51,52].

The primary objective of this work was to enhance the antifungal activity of medicarpin against *T. versicolor*, with broader implications for the development of natural product-based wood preservatives. Acetylation was specifically targeted based on its established role in wood protection technologies, where hydroxyl esterification of lignocellulosic components improves resistance to fungal and environmental degradation [53,54,55]. Accordingly, carbonyl-containing substituents were explored to evaluate whether analogous effects could be achieved through small-molecule derivatization.

Structural inspiration was also drawn from antifungal flavonoids such as alliodorin, which combines an aromatic core with carbonyl and aliphatic functionalities and has been reported to inhibit *T. versicolor* [56]. Based on this precedent, derivatives **6**–**9** were designed to incorporate aliphatic carbonyl moieties, while derivatives **2** and **3** were intended to assess whether antifungal activity depended on carbonyl functionality or could be retained with smaller, non-carbonyl substituents [57,58]. In contrast, derivatives **4** and **10**–**12** introduced bulkier aromatic groups, inspired by lignin precursors such as *p*-coumaryl, coniferyl, and sinapyl alcohols, under the hypothesis that mimicking these motifs could competitively interfere with fungal degradation pathways.

Biological assays revealed that, among all derivatives evaluated, only the acetylated derivative **5** exhibited enhanced antifungal activity relative to the parent compound **1**, achieving complete inhibition of *T. versicolor* at a lower concentration. To rationalize these observations, molecular docking and molecular dynamics simulations were conducted focusing on fungal laccase, a key oxidative enzyme implicated in lignocellulosic degradation and fungal virulence.

The inhibition of laccase primarily involves interactions with its catalytic copper centers—the type 1 (T1) site and the trinuclear copper cluster (TNC)—and their coordinating residues. In the *T. versicolor* laccase structure (PDB: 1GYC), the T1 copper is coordinated by two histidines (His458, His395) and a cysteine (Cys453), and is flanked by non-polar residues such as Ile455 and Phe463 [59,60]. His458 is functionally critical, forming part of the His458-Cys453-His359 triad that establishes the trigonal coordination geometry of the T1 site. This triad facilitates substrate oxidation and channels electrons to the TNC [61,62].

The TNC comprises a type 2 (T2) and two type 3 (T3) copper ions, coordinated by eight histidine ligands (His63, His66, His109, His111, His398, His400, His452, His454). This cluster is the site for dioxygen binding and reduction to water [63,64,65,66,67].

**Figure 3:**
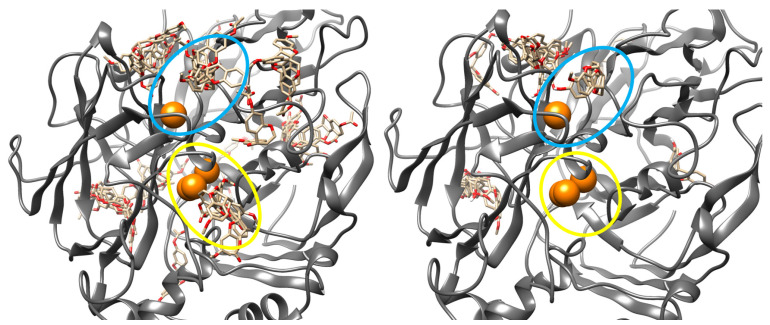
Docking poses of medicarpin (**1**) and its acetylated derivative (**5**) in *T. versicolor* laccase (PDB: 1GYC). Medicarpin (**1**, **right**) binds exclusively to the type 1 (T1) copper site (blue). In contrast, derivative **5** (**left**) demonstrates a dual binding mode, interacting with the T1 site and an additional site proximal to the trinuclear copper cluster (TNC, yellow). Copper ions are represented as orange spheres.

Docking simulations revealed distinct binding profiles for medicarpin (**1**) and its acetylated derivative (**5**). Medicarpin bound preferentially at the T1 site with a binding free energy (ΔG) of −7.57 kcal/mol. In contrast, derivative **5** exhibited stronger affinity for the same site (ΔG = −8.50 kcal/mol). Furthermore, derivative **5** displayed an additional, stable binding mode near the TNC (ΔG = −7.63 kcal/mol), an interaction not observed for the parent compound, although this pose occurred with lower conformational frequency in the docking ensemble (Figure 3).

**Figure 4:**
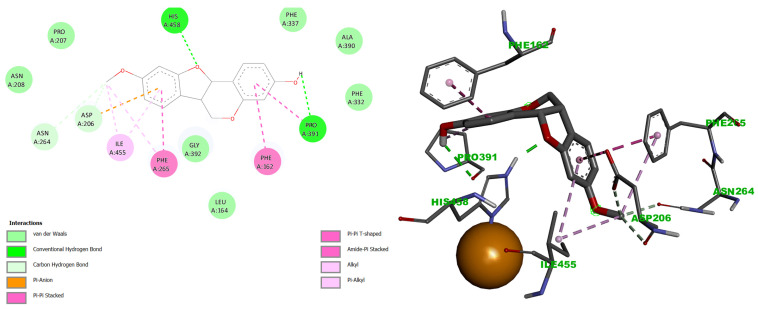
Docking interactions of medicarpin **1** with residues His458 and Pro391, by hydrogen bonds, at the CuT1 site (**left**). Additional contacts with surrounding residues (**right**). Visualized using BIOVIA Discovery Studio Visualizer. Similar colors indicate interactions within the same physicochemical category, distinguished by geometry and software-assigned labels.

Analysis of the docking interactions revealed that medicarpin (**1**) formed strong hydrogen bonds with His458 and Pro391, π-alkyl interactions with Ile455, Phe265, Phe162, and Pro391, π-anion interactions with Asp206 and Asn264, and van der Waals contacts with Leu164, Asn208, Pro207, Gly392, Phe337, Ala390, and Phe332 (Figure 4).

**Figure 5:**
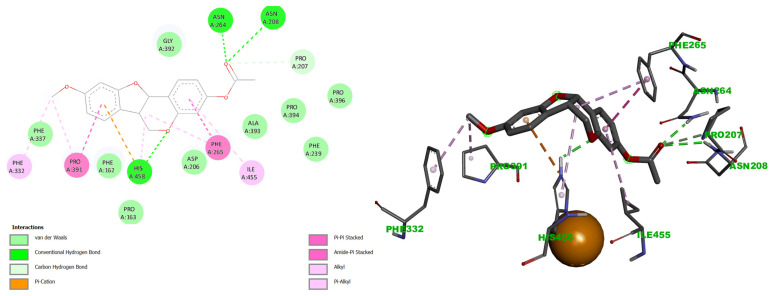
Docking interactions of the acetylated medicarpin derivative **5** at the CuT1 site. (**Left**): hydrogen bonds with His458, Asn264, and Asn208. (**Right**): π-alkyl interaction with Ile455 and π–anion interaction with Asp206. Visualized using BIOVIA Discovery Studio Visualizer. Similar colors indicate interactions within the same physicochemical category, distinguished by geometry and software-assigned labels.

Derivative **5** exhibited a distinct interaction profile at the T1 copper site, including hydrogen bonds with Asn264 and Asn208, π-alkyl interactions with Pro391, and van der Waals contacts with Pro163, Phe162, Asp206, Phe239, Phe337, Ala393, Pro394, and Pro396 (Figure 5).

Notably, both compounds shared interactions with His458, Pro391, Phe265, and Ile455, suggesting that derivative **5** adopts a different binding conformation than the parent compound. This conformational difference likely contributes to the observed enhancement in binding affinity (ΔG = −8.50 kcal/mol for **5** vs. −7.57 kcal/mol for **1**).

At the Cu T1 site, both ligands interacted with His458, a residue critical for maintaining the electrostatic environment of the T1 copper and facilitating electron transfer to the TNC [59,60]. However, derivative **5** adopted a distinct binding geometry, engaging additional residues involved in substrate orientation and access to the catalytic site. These interactions suggest that **5** may disrupt both electron transfer and substrate positioning more effectively than the parent compound, thereby impairing laccase activity to a greater extent.

**Figure 6:**
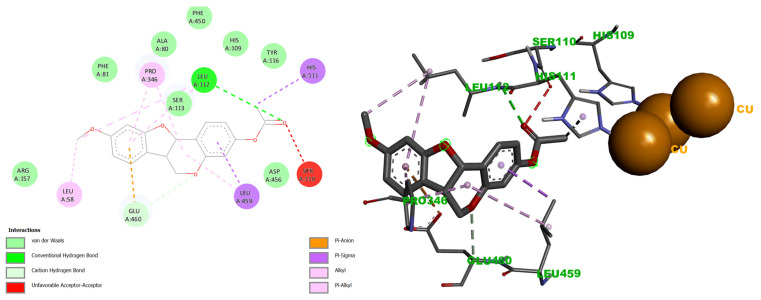
Docking interactions of the acetylated medicarpin derivative **5** at the TNC site. (**Left**): hydrogen bond with Leu112. (**Right**): π-σ interaction with His111 and van der Waals contact with His109. Visualized using BIOVIA Discovery Studio Visualizer. Similar colors indicate interactions within the same physicochemical category, distinguished by geometry and software-assigned labels.

Importantly, derivative **5** also formed transient interaction near the trinuclear copper cluster (TNC), including a hydrogen bond with Leu112, π-σ interactions with His111 and Leu459, π-alkyl contacts with Leu58 and Pro346, van der Waals interactions with Ala80, Phe81, His109, Ser113, Tyr116, Arg157, Phe450 and Asp456 (Figure 6). An unfavorable interaction with Ser110 was also observed. Notably, these residues are adjacent to the histidines that coordinate the T3 copper ions. Although this binding mode exhibited lower stability during molecular dynamics simulations, the proximity of **5** to the TNC suggests a secondary inhibitory mechanism that may further perturb electron transfer processes. In contrast, medicarpin (**1**) did not exhibit detectable interactions at the TNC site.

While most laccase inhibition studies emphasize interactions at the Cu T1 site, these results indicate that simultaneous or sequential engagement of regions surrounding the TNC may contribute to enhanced inhibitory efficacy [63,67].

Both medicarpin (**1**) and derivative **5** engaged His458 at T1 copper site, albeit through distinct interaction modes. Compound **1** formed a single hydrogen bond with the oxygen of the C-ring, whereas **5** established hydrogen bonding with the B-ring, π-cation interactions with the D-ring, and additional π-alkyl interactions with the B-ring. His458 is a key residue for substrate oxidation, providing electrostatic stability and facilitating electron transfer to the TNC [66,68]. Therefore, the interactions of **1** and **5** with His458 may disrupt redox potential and electron transfer efficiency.

Notably, derivative **5** exhibited additional interactions near the TNC, including contacts with His109 and His111 residues that coordinate the T3 cooper ions. These interactions suggest potential destabilization of the TNC and further inhibition of electron transfer [69] (Figure 6). While the literature has largely focused on T1 site interactions for laccase inhibition, these results indicate that effective inhibitors may require engagement with both T1 site and residues surrounding the TNC. This dual-site binding mode likely accounts for the enhanced antifungal activity of derivative **5** relative to the parent compound observed in *T. versicolor* assays.

Molecular dynamics simulations corroborated the docking results, demonstrating that derivative **5** remains stably anchored at the T1 copper site throughout the 80 ns trajectory, despite local flexibility of the binding pocket. Persistent hydrogen bonding and hydrophobic interactions with key residues involved in substrate guidance and electron transfer were observed, supporting a dual inhibitory mechanism that combines electrostatic perturbation and steric interference. In contrast, binding near the TNC was less stable, consistent with the lower conformational frequency observed for this site in the docking ensemble.

For the protein–ligand complex at the T1 copper site, the protein backbone exhibited an average RMSD of 3.324 Å, with fluctuations reaching up to 4.641 Å, indicating significant structural reorganization and conformational flexibility within the binding pocket (Figure S38). In contrast, the ligand (derivative **5**) remained comparatively stable, displaying a low average RMSD of 0.769 Å and maximum fluctuations of 1.501 Å. This behavior suggests that, although the T1 binding pocket undergoes dynamic rearrangements, the ligand maintains a stable binding pose throughout the simulation. Rather than being displaced, derivative **5** effectively accommodates the protein’s conformational flexibility, underscoring the robustness of the interaction at this site.

This stability can be attributed to the formation of strong and persistent interactions between derivative **5** and several residues within the T1 copper site environment. Hydrogen bonds were observed with Asn264 (124.24%), His458 (57.52%), Gly392 (90.66%), Phe265 (145.96%), Ile455 (176.90%), Asn208 (86.16%), Pro394 (248.70%), and Leu164 (147.13%). Among these, the interaction with His458 is particularly relevant, as this residue plays a central role in maintaining the electrostatic environment of the T1 copper center and mediating electron transfer during the laccase redox cycle [65,68,70]. The persistent engagement of His458 therefore suggests direct and sustained perturbation of electron flow, which is critical for enzymatic inactivation.

Notably, the interaction with Ile455, although not frequently highlighted in the literature, is also significant. Ile455 is positioned adjacent to residues surrounding the T1 copper center [65], and its engagement by the ligand indicates an extended interaction network that stabilizes derivative **5** within the binding pocket. This observation supports the notion that ligand anchoring at CuT1 is not limited to a single hotspot but involves a broader region of the substrate-binding cavity.

Additional interactions with Asn264, Phe265, Asn208, and Pro394 further reinforce this interpretation. Previous studies have identified these residues as key contributors to substrate orientation and channeling toward the T1 copper reactive site, where oxidation is initiated and electrons are subsequently transferred via His458 [65,71]. The strong interactions formed between derivative **5** and these residues suggest that, beyond electrostatic disruption near the copper center, the ligand may sterically hinder proper substrate accommodation and guidance. Consequently, inhibition is likely achieved through a combination of spatial obstruction and interference with the electron-transfer machinery of the enzyme.

In contrast, the protein–ligand complex formed near the TNC exhibited reduced stability. The protein backbone showed an average RMSD of 3.216 Å, with fluctuations reaching 4.251 Å, again indicating conformational rearrangements during the simulation. The ligand displayed an average RMSD of 0.947 Å with fluctuations up to 1.852 Å, suggesting transient positioning rather than stable anchoring (Figure S39). This instability is attributed to the formation of hydrogen bonds with low occupancy, likely resulting from electrostatic repulsion arising from the proximity of the copper ions within the TNC, as well as steric constraints inherent to this site.

These MD results are consistent with the molecular docking analysis, which showed a low population of ligand binding poses near the TNC. Both methods converge in identifying similar interacting residues at this site, including Leu112 (186.36%), Ser113 (71.14%), Leu58 (175.05%), Ala80 (177.28%), Pro86 (183.18%), Ser60 (199.6%), Leu494 (111.25%), and Gln499 (169.44%). Despite the reduced stability of this binding mode, its relevance should not be dismissed due to the close proximity of these residues to the copper atoms of the TNC.

In particular, Ser113 and Leu112 are directly associated with His111, a residue coordinating the copper ions within the TNC [72]. Even transient interactions between derivative **5** and these residues may induce local electrostatic perturbations analogous to those observed at the CuT1 site at His458, potentially contributing to partial disruption of the catalytic cycle.

Residues exhibiting occupancy values exceeding 100% (e.g., Asn208, Pro394, Leu112, Gln499) indicate multiple, temporally distinct interaction events occurring during the simulation, with cumulative occupancy reflecting repeated contact formation. Although a larger number of ligand-residue interactions were detected at both the T1 and TNC sites, many displayed lower occupancy values, as summarized in Table S1.

Binding free energy calculations using the MM-GBSA approach further supported these findings, yielding more favorable binding energies for derivative **5** at the CuT1 site, with a binding energy of −29.07 kcal/mol, compared to compound **1**, where the binding energy at CuT1 was −24.43 kcal/mol, indicating that binding with derivative **5** is more favored. Furthermore, the transient (reduced) interaction of derivative **5** at the TNC site demonstrated a binding energy of −23.69 kcal/mol.

Collectively, these results indicate that the enhanced antifungal activity of derivative **5** is primarily attributable to the introduction of the acetyl substituent into the parent medicarpin structure. This modification alters the physicochemical properties of the molecule, increasing its hydrophobic character and improving compatibility with the predominantly hydrophobic substrate-binding cavity of laccase. Docking results consistently showed more favorable binding energies for derivative **5** than for the parent compound (**1**) across the evaluated binding pockets.

Although only derivatives **1** and **5** are discussed in detail here, docking studies were performed for the entire series. Ether derivatives **2**–**4** showed binding within the substrate cavity for more than 75% of their conformers, with binding energies ranging from −8.85 to −7.5 kcal/mol. This behavior suggests that these compounds may act as laccase substrates rather than inhibitors, consistent with their reduced antifungal activity observed experimentally.

Similarly, derivatives designed as lignin precursors (**10**–**12**) were initially hypothesized to enhance catalytic-site interactions; however, inhibition assays revealed diminished activity. Docking analysis indicates that, although these ligands could approach the T1 copper site, they failed to penetrate deeply or establish strong interactions with key residues, likely due to steric hindrance and unfavorable electronic effects from their aromatic substituents. Aliphatic ester derivatives (**6**–**9**) exhibited binding populations below 10% of the total analyzed conformers, correlating with their poor antifungal activity against *T. versicolor*. All results, including binding energy (kcal/mol), interaction frequencies (%), and ligand–amino acid residue interactions of medicarpin (**1**) and derivatives (**2**–**12**) with T1 and TNC, can be viewed in Table S2.

In summary, functionalization of the hydroxyl group in medicarpin with an acetyl moiety yields a derivative with improved affinity for laccase catalytic sites, enhanced interaction persistence, and increased capacity to disrupt both electrostatic and steric components of the enzymatic mechanism. This dual mode of interference―perturbation of electron transfer coupled with obstruction of substrate accommodation―provides a coherent molecular explanation for the increased antifungal efficacy of derivative **5** and underscores the potential of rational modification of natural products for wood preservation applications.

## 4. Materials and Methods

### 4.1. Plant Material and Isolation of Medicarpin

Heartwood from *Andira inermis* was collected in Taretán, Michoacán, México, and taxonomically authenticated at the Faculty of Wood Engineering, Universidad Michoacana de San Nicolás de Hidalgo. The air-dried material was milled and exhaustively macerated in ethyl acetate at room temperature for seven days. The solvent was removed under reduced pressure to obtain a crude extract, from which medicarpin (**1**) was isolated by standard chromatographic procedures.

### 4.2. Instrumentation and Analysis

^1^H and ^13^C NMR spectra were recorded on a Varian Mercury Plus spectrometer operating at 400 MHz and 101 MHz (Varian, Inc., Palo Alto, California, United States), respectively. Analytical HPLC was performed using an AD-H chiral column (Daicel Chemical Industries, Ltd., Minato-Ku, Tokyo, Japan). Melting points were determined with a Fischer model 1237 apparatus (Thermo Fischer Scientific, Waltham, Massachusetts, United States) and are uncorrected.

### 4.3. Antifungal Activity Assay

The antifungal activity was evaluated against *T. versicolor* (ATCC 32745) using potato dextrose agar (3.9%). Medicarpin derivatives were incorporated into the culture medium at concentrations of 100, 150, and 200 mg/L prior to solidification. Plates were inoculated with mycelial plugs and incubated at 28 ± 2 °C for seven days. Fungal growth inhibition was calculated relative to untreated controls using standard percentage inhibition criteria [21,56]

$$\%\;inhibition=\left(\frac{growth\;control-growth\;treatment}{growth\;control\;}\right)\times 100$$

All experiments were performed in triplicate, and the results are expressed as the mean ± standard deviation (SD). Statistical analysis was conducted using GraphPad Prism (version 9; GraphPad Software, San Diego, CA, USA). Differences in antifungal activity among compounds and concentrations were evaluated using one-way analysis of variance (ANOVA), followed by Tukey’s post hoc multiple comparison test. Statistical significance was determined at *p* < 0.05.

### 4.4. Molecular Docking Studies

The three-dimensional structures of medicarpin (**1**) and derivatives **2**–**12** were geometry-optimized using Gaussian 16 at the DFT B3LYP/6-311** level. The crystal structure of *T. versicolor* laccase (PDB ID: 1GYC) [59] was prepared by adding polar hydrogens and Kollman charges. Docking simulations were carried out with AutoDock 4.2 employing the Lamarckian Genetic Algorithm [73]. A grid box (126 × 126 × 126 points―0.375 Å spacing) encompassing the catalytic region (Cu T1 and TNC) was defined, and 1000 binding poses were generated per ligand. Copper parameters were manually assigned according to the instructions in the AutoDock 4.2 User Gude, and binding modes were analyzed using BIOVIA Discovery Studio Visualizer v21.1.0.20298.

### 4.5. Molecular Dynamics Simulation

Molecular dynamics (MD) simulations were performed on the highest-scoring ligand–protein complexes identified by molecular docking. Ligand parameters were assigned using the General AMBER Force Field (GAFF) via the Antechamber module, with missing parameters assigned by Parmchk. The protein was modeled with the AMBER ff14SB force field [74,75]. The cooper ions were restrained to metal–residue bonds through bond distances and angles with their coordinating residues (histidines); these parameters were derived from the 1GYC crystal structure.

Each solvated system was constructed by placing the complex in a rectangular periodic box (126 × 126 × 126 Å) with a 20 Å buffer of TIP3P water molecules. System neutrality was achieved by adding Na^+^ and Cl^−^ counterions to a physiological ionic concentration of 0.15 M. All simulations were executed using the AMBER 20 package [76].

An energy minimization of 80,000 steps using the conjugate gradient algorithm was performed prior to dynamic equilibration. The systems were then gradually heated from 0 to 310 K over 50 ps under the NVT ensemble, followed by a 100 ps equilibration under NPT conditions at 1 atm and 310 K. Temperature was regulated with a Langevin thermostat, and production simulations were carried out for 80 ns with a 2 fs integration time step.

Trajectories were analyzed for stability by calculating the root-mean-square deviation (RMSD) of the protein backbone and ligand. Hydrogen bond occupancies were determined using geometric criteria of a 3.5 Å donor-acceptor distance and a 120° angle cutoff, as implemented in CPPTRAJ [77]. The binding free energy for each complex was estimated using the Molecular Mechanics-Generalized Born Surface Area (MM/GBSA) method within the AMBER framework [78].

### 4.6. Isolation and Synthesis of Medicarpin Derivatives

Detailed information for this section, along with the NMR spectra, is provided in the Supporting Information.

## 5. Conclusions

In this study, we demonstrated that the biological activity of a natural product can be significantly enhanced through small and rational structural modifications without altering its core scaffold, providing a simple and reproducible strategy for the design and development of antifungal agents of natural origin. This objective was achieved through acetylation of the phenolic hydroxyl group of medicarpin, resulting in a marked increase in antifungal activity against the white-rot fungus *T. versicolor*. Compared to native medicarpin, which inhibits fungal growth at 150 mg/L, the acetylated derivative achieved complete inhibition at 100 mg/L, corresponding to an improvement of approximately 33% in biological efficacy.

To elucidate the molecular determinants underlying the enhanced antifungal activity of the acetylated derivative, experimental findings were complemented by molecular docking and molecular dynamics simulations targeting laccase, the primary oxidative enzyme secreted by *T. versicolor* and a key mediator of nutrient acquisition and fungal survival. While the interaction of native medicarpin with the catalytic T1 copper site of laccase has been previously described, the acetylated derivative exhibited more favorable binding energies and stabilizing interactions at this site, leading to more efficient interference with the enzyme’s redox processes and subsequent inactivation. Notably, an additional binding mode for the acetylated derivative was identified proximal to the trinuclear copper cluster (TNC)—a region not previously considered relevant for inhibition. This ancillary interaction may contribute complementarily to the disruption of electron transfer during catalysis.

Collectively, these results provide mechanistic insight into how rational structural modification enhances binding at the catalytic copper centers of laccase, resulting in more efficient enzyme inactivation and fungal inhibition at lower concentrations. This study establishes the acetylated medicarpin derivative as a promising antifungal candidate and underscores the potential of modifying natural products for the development of wood preservatives and antifungal formulations. Future work should focus on determining key pharmacodynamic parameters—particularly the MIC—and evaluating suitable formulation and dispersion methods to advance this derivative toward a practical preservative product.

## Figures and Tables

**Table 1:** Reaction conditions used for the synthesis of medicarpin derivatives (**2**–**12**).

	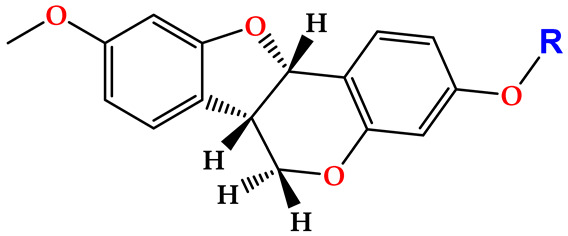 Medicarpin Derivatives						
R		Yield (%)	Base	Solvent	Temperature	Time (h)	Atm
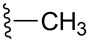	(**2**)	85	NaOH	THF	r.t.	24	Air
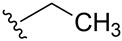	(**3**)	75	NaOH	THF	r.t.	24	Air
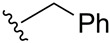	(**4**)	60	NaOH	THF	r.t.	24	Air
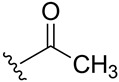	(**5**)	95	Py	Py	r.t.	24	Air
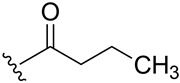	(**6**)	80	Py	Py	Reflux	3	N_2_
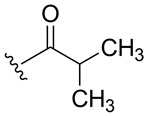	(**7**)	80	Py	Py	Reflux	3	N_2_
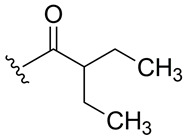	(**8**)	65	Py	Py	Reflux	3	N_2_
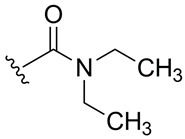	(**9**)	60	Py	Py	Reflux	3	N_2_
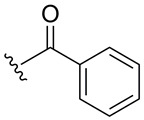	(**10**)	60	TEA	THF (Anh)	r.t.	24	N_2_
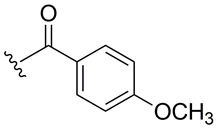	(**11**)	70	Py	THF (Anh)	r.t.	24	N_2_
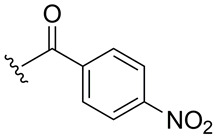	(**12**)	80	TEA	THF (Anh)	r.t.	24	N_2_

THF: tetrahydrofuran; Py: pyridine; TEA: triethyl amine; r.t.: room temperature.

**Table 2:** Diagnostic ^1^H and ^13^C NMR signals confirming functionalization of medicarpin derivatives (**2**–**12**).

Compound	Functional Group Introduced	Diagnostic ^1^H NMR Signals (δ in ppm, *J* in Hz)	Diagnostic ^13^C NMR Signals (δ in ppm)
**2**	Methyl ether	3.79 (s, 3H, O-CH_3_)	-
**3**	Ethyl ether	4.01 (q, J = 7.0 Hz, 2H, O-CH_2_) 1.40 (t, J = 7.0 Hz, 3H, CH_3_)	-
**4**	Benzyl ether	5.05 (s, 2H, O-CH_2_) 7.30–7.40 (m, 5H, Ar-H)	-
**5**	Acetate	2.30 (s, 3H, O-C(O)CH_3_)	169.1 ppm (C=O)
**6**	Butyrate	1.04 (t, J = 7.4, 3H, CH_3_) 1.78 (h, J = 7.5, 2H, CH_2_) 2.53 (t, J = 7.4, 2H, CH_2_)	171.8 (C=O)
**7**	Isobutyrate	1.31 (d, J = 7.0, 6H, CH_3_) 2.79 (hept, J = 7.0, 1H, CH)	175.3 (C=O)
**8**	2-Ethylbutanoate	1.01 (t, J = 7.4, 6H, CH_3_) 1.65–1.76 (m, 4H, CH_2_) 2.44 (m, 1H, CH)	174.3 (C=O)
**9**	Diethylcarbamate	1.22 (m, 6H, CH_3_) 3.40 (m, 4H, CH_2_)	153.7 (C=O)
**10**	Benzoate	7.52 (t, J = 7.7, 2H, Ar-H) 7.65 (t, J = 7.5, 2H, Ar-H) 8.20 (d, J = 8.5, 1H, Ar-H)	1.64.8 (C=O)
**11**	4-Methoxybenzoate	3.90 (s, 3H, O-CH_3_) 6.99 (d, J = 8.7, 2H, Ar-H) 8.15 (d, J = 8.6, 2H, Ar-H)	164.5 (C=O)
**12**	4-Nitrobenzoate	8.38 (m, 4H, Ar-H)	163.0 (C=O)

## Data Availability

All data generated or analyzed during this study are included in this article and its Supplementary Materials Files.

## Supplementary Materials

The following supporting information can be downloaded at: https://www.mdpi.com/article/10.3390/ijms27062878/s1.
