# Supplementary material for: Enhanced Inhibition of Trametes versicolor by Structurally Modified Medicarpin: In Vitro Evaluation and In Silico Insights into Laccase Binding
Source: Int J Mol Sci. 2026 Mar 22;27(6):2878. doi: 10.3390/ijms27062878 (PMC13026702; doi:10.3390/ijms27062878)
Supplement: Supplementary file 1 [file ijms-27-02878-s001.zip › ijms-4175916-supplementary.pdf]

Enhanced Inhibition of *Trametes versicolor* by Structurally Modified Medicarpin: *In Vitro* Evaluation and I*n Silico* Insights into Laccase Binding

Santiago José Guevara-Martínez [1], José Domingo Rivera-Ramírez [1], Rebeca Escutia-Gutierrez [2], Marco Antonio Pérez-Cisneros [3], Francisco Villanueva-Mejía [4], Stephanie García-Zavala [5], Rafael Herrera-Bucio [5,*] and Fredy Geovannini Morales-Palacios [5,]**

[1] Laboratorio de Química Farmacéutica, Departamento de Farmacobiología, Centro Universitario de Ciencias Exactas e Ingenierías, Universidad de Guadalajara, Boulevard Gral. Marcelino García Barragán 1421, Olímpica, Guadalajara 44430, Jalisco, Mexico
[2] Departamento de Biología Molecular y Genómica, Centro Universitario de Ciencias de la Salud, Universidad de Guadalajara, Sierra Mojada 950, Independencia Oriente, Guadalajara 44430, Jalisco, Mexico
[3] Departamento de Electrofotónica, Centro Universitario de Ciencias Exactas e Ingenierías, Universidad de Guadalajara, Boulevard General. Marcelino García Barragán 1421, Olímpica, Guadalajara 44430, Jalisco, Mexico
[4] Laboratorio de Herramientas Computacionales, Instituto Tecnológico de Pabellón de Arteaga, Carretera a la estación de Rincón de Romos, Km 1, Aguascalientes 20267, Aguascalientes, México
[5] Instituto de Investigación Químico Biológicas, Universidad Michoacana de San Nicolás de Hidalgo, Francisco J. Múgica, s/n, Morelia 58030, Michoacán, México; e-mail@e-mail.com
* Correspondence: rafael.herreta.bucio@umich.mx
** Principal correspondence: geovannini.morales@umich.mx

Supporting information

Tables of contents

1. General consideration 1
2. Isolation and synthesis of Medicarpin Derivatives 2
3. NMR spectra and HPLC chromatogram 5
4. RMSD molecular dynamics 30
5. Table H-bonds molecular dynamics 32
6. Docking results of derivatives 35

1.- General consideration

Column chromatography was performed using Merck Silica Gel (70-230 Mesh). ^{1}H and ^{13}C NMR spectra were recorded on a Varian Mercury Plus spectrometer operating at 400 MHz and 101 MHz, respectively. Analytical HPLC was performed using an AD-H chiral column. Melting points were determined with an EVEL model 1237 apparatus and are uncorrected.

2.- Isolation and Synthesis of Medicarpin Derivatives

Parent compound: Medicarpin

(6a*S*,11a*S*)-9-methoxy-6a,11a-dihydro-6H-benzofuro[3,2-*c*]chromen-3-ol (**1**)

Medicarpin (**1**) was purified by column chromatography (silica gel, 70-230 ASTM, Whatman) using n-hex/EtOAc 95:5 as eluent. White amorphous crystals were obtained (mp 117-118 °C). Complete ^{1}H and ^{13}C NMR assignments were as follows:

^{1}H NMR (400 MHz, $CDCl_3$) δ (ppm): 3.52 (ddd, J = 10.2, 7.5, 4.1 Hz, 1H), 3.62 (t, J = 10.9 Hz, 1H), 3.77 (s, 3H), 4.24 (dd, J = 10.9, 5.0 Hz, 1H), 5.07 (s, 1H), 5.50 (d, J = 6.7 Hz, 1H), 6.41 (d, J = 2.5 Hz, 1H), 6.45 (s, 1H), 6.46 (dd, J = 5.8, 2.1 Hz, 1H), 6.55 (dd, J = 8.4, 2.5 Hz, 1H), 7.13 (d, J = 8.7 Hz, 1H), 7.39 (d, J = 8.4 Hz, 1H).

^{13}C NMR (101 MHz, $CDCl_3$) δ (ppm): 39.4, 55.4, 66.5, 78.5, 96.8, 103.6, 106.3, 109.7, 112.6, 119.0, 124.7, 132.1, 156.6, 156.9, 160.6, 161.0.

Eleven derivatives (**2**-**12**) were synthesized from 50 mg (0.185 mmol) of **1** each via standard etherification, acetylation or acylation reactions, employing NaOH, pyridine, or triethylamine as bases and the corresponding alkyl halides or acid anhydrides. Products were purified by column chromatography or recrystallization, and their structures were confirmed by ^{1}H and ^{13}C NMR spectroscopy.

Methyl Ether Derivative of Medicarpin

(6a*S*,11a*S*)-3,9-dimethoxy-6a,11a-dihydro-6H-benzofuro[3,2-*c*]chromene (**2**)

Medicarpin methyl ether (**2**) was synthesized by first dissolving 100 mg NaOH in 2 mL THF. To this solution, 0.401 mmol methyl iodide was added, and the mixture was stirred at room temperature for 24 h. The reaction mixture was purified by column chromatography using an n-hex/EtOAc 98:2 eluent system, resulting in an 85% yield (44.7 mg) of white amorphous crystals (mp 131-132 °C). NMR data were:

^{1}H NMR (400 MHz, $CDCl_3$) δ (ppm): 3.50 (m, 1H), 3.64 (m, 1H), 3.76 (s, 3H), 3.79 (s, 3H), 4.23 (m, 1H), 5.48 (d, J = 7.0 Hz, 1H), 6.72 (s, 1H), 6.43 (s, 1H), 6.45 (dd, J = 6.8, 1.7 Hz, 1H), 6.63 (dd, J = 8.5, 2.6 Hz, 1H), 7.13 (d, J = 8.8 Hz, 1H), 7.40 (d, J = 8.7 Hz, 1H).

^{13}C NMR (101 MHz, $CDCl_3$) δ (ppm): 39.5, 55.4, 55.3, 66.5, 78.4, 93.7, 104.6, 106.3, 109.2, 112.3, 117.8, 124.6, 131.7, 148.0, 156.5, 160.6, 160.9.

Ethyl Ether Derivative of Medicarpin

(6a*S*,11a*S*)-3-ethoxy-9-methoxy-6a,11a-dihydro-6H-benzofuro[3,2-*c*]chromene (**3**)

Medicarpin ethyl ether (**3**) was synthesized by first dissolving 100 mg NaOH in 2 mL THF. To this solution, 0.375 mmol ethyl bromide was added, and the mixture was stirred at room temperature for 24 h. The reaction mixture was purified by column chromatography using an n-hex/EtOAc 98:2 eluent system, resulting in an 75% yield (41.4 mg) of laminar crystals (mp 113-115 °C). NMR data were:

^{1}H NMR (400 MHz, $CDCl_3$) δ (ppm): 1.40 (t, J = 7.0 Hz, 3H), 3.50 (m, 1H), 3.63 (m, 1H), 3.77 (s, 3H), 4.01 (q, J = 7.0 Hz, 2H), 4.24 (m, 1H), 5.48 (d, J = 6.9 Hz, 1H), 6.72 (s, 1H), 6.43 (s, 1H), 6.45 (m, 1H), 6.62 (dd, J = 8.5, 2.6 Hz, 1H), 7.13 (d, J = 8.8 Hz, 1H), 7.39 (d, J = 8.6 Hz, 1H).

^{13}C NMR (101 MHz, $CDCl_3$) δ (ppm): 14.7, 40.1, 55.4, 63.5, 66.4, 78.5, 93.7, 104.6, 106.3, 109.6, 112.4, 117.9, 124.7, 131.6, 148.0, 156.4, 160.3, 161.9.

Benzyl Ether Derivative of Medicarpin

(6a*S*,11a*S*)-3-(benzyloxy)-9-methoxy-6a,11a-dihydro-6H-benzofuro[3,2-*c*]chromene (**4**)

Medicarpin benzyl ether (**4**) was synthesized by first dissolving 100 mg NaOH in 2 mL THF. To this solution, 0.211 mmol benzyl bromide was added, and the mixture was stirred at room temperature

for 24 h. The reaction mixture was purified by column chromatography using an n-hex/EtOAc 98:2 eluent system, resulting in an 60% yield (40.1 mg) of amorphous crystals (mp 100-101 °C). NMR data were:

^{1}H NMR (400 MHz, $CDCl_3$) δ (ppm): 3.50 (m, 1H), 3.63 (m, 1H), 3.76 (s, 3H), 4.23 (m, 1H, H), 5.05 (s, 2H), 5.51 (d, J = 6.8 Hz, 1H), 6.71 (dd, J = 8.1, 3.1 Hz, 1H), 6.43 (s, 1H), 6.45 (dd, J = 4.3, 2.1 Hz, 1H), 6.55 (d, J = 2.2 Hz, 1H), 7.13 (d, J = 8.8 Hz, 1H), 7.33 (d, J = 7.0 Hz, 1H), 7.40 (m, 5H).

^{13}C NMR (101 MHz, $CDCl_3$) δ (ppm): 39.5, 55.4, 66.5, 70.0, 78.5, 93.8, 104.6, 106.3, 109.8, 112.6, 117.8, 124.7, 127.4, 127.9, 128.5, 131.8, 136.6, 148.0, 156.5, 160.1, 160.6.

Acetylated Derivative of Medicarpin

(6a*S*,11a*S*)-9-methoxy-6a,11a-dihydro-6H-benzofuro[3,2-*c*]chromen-3-yl acetate (**5**)

Medicarpin acetate (**5**) was obtained by reacting 0.5 mL of pyridine with 0.278 mmol of acetic anhydride at room temperature for 24 h. The reaction mixture was purified by column chromatography using an n-hex/EtOAc 95:5 eluent system, resulting in an 95% yield (54.9 mg) of needle-like crystals (mp 105-106 °C). NMR data were:

^{1}H NMR (400 MHz, $CDCl_3$) δ (ppm): 2.30 (s, 3H), 3.56 (ddd, J = 10.2, 7.2, 3.8 Hz, 1H), 3.63 (t, J = 9.2 Hz, 1H), 3.77 (s, 3H), 4.27 (dd, J = 10.7, 4.8 Hz, 1H), 5.52 (d, J = 6.6 Hz), 6.45 (s, 1H), 6.47 (d, J = 2.3 Hz, 1H), 6.71 (d, J = 2.3 Hz, 1H), 6.80 (dd, J = 8.4, 2.3 Hz, 1H), 7.13 (dd, J = 8.6, 0.7 Hz, 1H), 7.54 (d, J = 8.6 Hz, 1H).

^{13}C NMR (101 MHz, $CDCl_3$) δ (ppm): 21.0, 39.4, 55.4, 66.5, 78.0, 96.8, 106.5, 110.7, 115.2, 117.7, 118.7, 124.7, 131.7, 151.5, 156.1, 160.5, 161.1, 169.1.

Butyrate Derivative of Medicarpin

(6a*S*,11a*S*)-9-methoxy-6a,11a-dihydro-6H-benzofuro[3,2-*c*]chromen-3-yl butyrate (**6**)

Medicarpin acetate (**6**) was obtained by reacting 2 mL of pyridine with 0.555 mmol of butyryl chloride under reflux in a nitrogen atmosphere for 3 h. The reaction mixture was purified by column chromatography using an n-hex/EtOAc 95:5 eluent system, resulting in an 80% yield (50.6 mg) of a yellow oil. NMR data were:

^{1}H NMR (400 MHz, $CDCl_3$) δ (ppm): 1.04 (t, J = 7.4 Hz, 3H), 1.78 (h, J = 7.5 Hz, 2H), 2.53 (t, J = 7.4 Hz, 2H), 3.54 (m, 1H), 3.65 (t, J = 10.9 Hz, 1H), 3.77 (s, 3H), 4.26 (dd, J = 11.2, 4.6 Hz, 1H), 5.50 (d, J = 7.0 Hz, 1H), 6.71 (d, J = 10.3 Hz, 1H), 6.44 (s, 1H), 6.46 (dd, J = 7.4, 1.9 Hz, 1H), 6.78 (dd, J = 8.4, 2.4 Hz, 1H), 7.14 (d, J = 9.1 Hz, 1H), 7.51(d, J = 8.4 Hz, 1H).

^{13}C NMR (101 MHz, $CDCl_3$) δ (ppm): 13.5, 18.3, 36.1, 40.1, 55.4, 66.5, 78.0, 93.7, 110.6, 104.6, 115.3, 117.6, 118.7, 124.7, 131.6, 151.6, 156.1, 160.5, 161.1, 171.8.

Isobutyrate Derivative of Medicarpin

(6a*S*,11a*S*)-9-methoxy-6a,11a-dihydro-6H-benzofuro[3,2-*c*]chromen-3-yl isobutyrate (**7**)

Medicarpin isobutyrate (**7**) was obtained by reacting 2 mL of pyridine with 0.555 mmol of isobutyryl chloride under reflux in a nitrogen atmosphere for 3 h. The reaction mixture was purified by column chromatography using an n-hex/EtOAc 95:5 eluent system, resulting in an 80% yield (50.6 mg) of needle-like crystals (mp 70-72 °C). NMR data were:

^{1}H NMR (400 MHz, $CDCl_3$) δ (ppm): 1.31 (d, J = 7.0 Hz, 6H), 2.79 (h, J = 7.0 Hz, 1H), 3.54 (m, 1H), 3.65 (t, J = 10.9 Hz, 1H), 3.77 (s, 3H), 4.26 (dd, J = 11.1, 4.9 Hz, 1H), 5.50 (d, J = 7.0 Hz, 1H), 6.71 (d, J = 13.5 Hz, 1H), 6.44 (s, 1H), 6.46 (dd, J = 6.8, 2.1 Hz, 1H), 6.78 (dd, J = 8.4, 2.5 Hz, 1H), 7.14 (d, J = 8.9 Hz, 1H), 7.50 (d, J = 8.4 Hz, 1H).

^{13}C NMR (101 MHz, $CDCl_3$) δ (ppm): 18.8, 34.1, 39.8, 55.4, 66.5, 78.0, 93.8, 110.6, 104.6, 115.2, 117.5, 118.7, 124.7, 131.6, 151.8, 156.1, 160.5, 161.1, 175.3.

2-Ethylbutanoate Derivative of Medicarpin

(6a*S*,11a*S*)-9-methoxy-6a,11a-dihydro-6H-benzofuro[3,2-*c*]chromen-3-yl 2-ethylbutanoate (**8**)

Medicarpin 2-ethylbutyrate (**8**) was obtained by reacting 2 mL of pyridine with 0.555 mmol of ethylbutyryl chloride under reflux in a nitrogen atmosphere for 3 h. The reaction mixture was purified by column chromatography using an n-hex/EtOAc 95:5 as eluent system, resulting in an 65% yield (42.8 mg) of a colorless translucent oil. NMR data were:

^1H NMR (400 MHz, $CDCl_3$) δ (ppm): 1.01 (t, J = 7.4 Hz, 6H), 1.65 (m, 2H), 1.76 (m, 2H), 2.44 (m, 1H), 3.53 (m, H1), 3.64 (m, 1H), 3.76 (s, 3H), 4.26 (m, 1H), 5.51 (d, J = 6.8 Hz, 1H), 6.70 (d, J = 10.5 Hz, 1H), 6.43 (s, 1H), 6.46 (dd, J = 6.8, 2.2 Hz, 1H), 6.77 (dd, J = 5.7, 2.6 Hz, 1H), 7.13 (d, J = 9.0 Hz, 1H), 7.53 (d, J = 8.4 Hz, 1H).

^{13}C NMR (101 MHz, $CDCl_3$) δ (ppm): 11.7, 25.0, 48.8, 40.1, 55.4, 66.4, 77.9, 93.7, 110.7, 104.6, 115.3, 117.5, 118.7, 124.7, 131.6, 151.7, 156.1, 160.5, 161.1, 174.3.

Diethylcarbamate Derivative of Medicarpin

(6a*S*,11a*S*)-9-methoxy-6a,11a-dihydro-6H-benzofuro[3,2-*c*]chromen-3-yl diethylcarbamate (**9**)

Medicarpin diethylcarbamate (**9**) was obtained by reacting 2 mL of pyridine with 0.555 mmol of diethylcarbamoyl chloride under reflux in a nitrogen atmosphere for 3 h. The reaction mixture was purified by column chromatography using an n-hex/EtOAc 95:5 as eluent system, resulting in an 60% yield (41.2 mg) of needle-like crystals (mp 75-76 °C). NMR data were:

^1H NMR (400 MHz, $CDCl_3$) δ (ppm): 1.22 (m, 6H), 3.40 (m, 4H), 3.56 (m, 1H), 3.63 (m, 1H), 3.77 (s, 3H), 4.27 (dd, J = 10.6, 4.6 Hz, 1H), 5.52 (d, J = 6.5 Hz, 1H), 6.74 (d, J = 2.3 Hz, 1H), 6.45 (s, 1H), 6.46 (dd, J = 5.7, 2.3 Hz, 1H), 6.84 (dd, J = 8.4, 2.3 Hz, 1H), 7.14 (d, J = 8.8 Hz, 1H), 7.51 (d, J = 8.4 Hz, 1H).

^{13}C NMR (101 MHz, $CDCl_3$) δ (ppm): 13.3, 14.1, 41.8, 42.2, 39.5, 55.4, 66.5, 78.1, 96.8, 110.6, 106.4, 115.5, 116.9, 118.8, 124.7, 131.4, 152.5, 156.0, 160.5, 161.0, 153.7.

Benzoate Derivative of Medicarpin

(6a*S*,11a*S*)-9-methoxy-6a,11a-dihydro-6H-benzofuro[3,2-*c*]chromen-3-yl benzoate (**10**)

Medicarpin benzoate (**10**) was obtained by reacting 2 mL of anhydrous THF with 0.3 mL of triethylamine (TEA) as base, stirred for 24 h, at room temperature. The reaction mixture was purified by column chromatography using an n-hex/EtOAc 95:5 as eluent system, resulting in an 60% yield (41.6 mg) of needle-like crystals (mp 138-140 °C). NMR data were:

^1H NMR (400 MHz, $CDCl_3$) δ (ppm): 3.61 (m, 1H), 3.65 (t, J = 10.3 Hz, 1H), 3.79 (s, 3H), 4.31 (dd, J = 10.6, 4.6 Hz, 1H), 5.56 (d, J = 6.6 Hz, 1H), 6.85 (d, J = 2.3 Hz, 1H), 6.47 (s, 1H), 6.48 (d, J = 2.2 Hz, 1H), 6.94 (dd, J = 8.4, 2.3 Hz, 1H), 7.16 (d, J = 8.7 Hz, 1H), 7.52 (t, J = 7.7 Hz, 1H), 7.60 (d, J = 8.4 Hz, 1H), 7.65 (t, J = 7.5 Hz, 1H), 8.20 (d, J = 8.5 Hz, 1H).

^{13}C NMR (101 MHz, $CDCl_3$) δ (ppm): 39.5, 55.4, 66.6, 78.0, 96.9, 106.5, 110.8, 115.4, 117.8, 118.7, 124.7, 128.5, 129.3, 130.1, 131.8, 133.6, 151.9, 156.3, 160.5, 161.2, 164.8.

4-Methoxybenzoate Derivative of Medicarpin

(6a*S*,11a*S*)-9-methoxy-6a,11a-dihydro-6H-benzofuro[3,2-*c*]chromen-3-yl 4-methoxybenzoate (**11**)

Medicarpin 4-methoxybenzoate (**11**) was obtained by reacting 0.3 mL of pyridine as base, 2 mL of anhydrous THF as solvent, and 0.185 mmol of 4-methoxybenzoyl chloride and the mixture was

stirred at room temperature for 24 h. The reaction mixture was purified by column chromatography using an n-hex/EtOAc 95:5 as eluent system, resulting in an 70% yield (50.7 mg) of needle-like crystals (mp 160-162 °C). NMR data were:

^{1}H NMR (400 MHz, $CDCl_3$) δ (ppm): 3.61 (m, 1H), 3.66 (t, J = 10.7 Hz, 1H), 3.78 (s, 3H), 3.90 (s, 3H), 4.30 (dd, J = 10.9, 4.5 Hz, 1H), 5.56 (d, J = 6.6 Hz, 1H), 6.46 (s, 1H), 6.48 (d, J = 2.5 Hz, 1H), 6.83 (d, J = 2.1 Hz, 1H), 6.92 (dd, J = 8.4, 2.4 Hz, 1H), 6.99 (d, J = 8.7 Hz, 2H), 7.16 (d, J = 8.8 Hz, 1H), 7.58 (d, J = 8.4 Hz, 1H), 8.15 (d, J = 8.6 Hz, 2H).

^{13}C NMR (101 MHz, $CDCl_3$) δ (ppm): 39.5, 55.4, 66.5, 78.0, 96.8, 106.4, 110.9, 113.7, 115.5, 117.6, 118.7, 121.5, 124.7, 131.7, 132.3, 151.9, 156.2, 160.5, 161.1, 163.9, 164.5.

4-Nitrobenzoate Derivative of Medicarpin

(6a*S*,11a*S*)-9-methoxy-6a,11a-dihydro-6H-benzofuro[3,2-*c*]chromen-3-yl 4-nitrobenzoate (**12**)

Medicarpin 4-nitrobenzoate (**12**) was obtained by reacting 0.3 mL of triethylamine as base, 2 mL of anhydrous THF as solvent, and 0.185 mmol of 4-nitrobenzoyl chloride and the mixture was stirred at room temperature for 24 h. The reaction mixture was purified by column chromatography using an n-hex/EtOAc 95:5 as eluent system, resulting in an 8o% yield (57.8 mg) of needle-shaped crystals (mp 148-150 °C). NMR data were:

^{1}H NMR (400 MHz, $CDCl_3$) δ (ppm): 3.61 (m, 1H), 3.67 (dt, J = 11.1, 3.2 Hz, 1H), 3.79 (s, 3H), 4.31 (dd, J = 12.6, 5.6 Hz, 1H), 5.55 (dd, J = 9.8, 7.0 Hz, 1H), 6.48 (s, 1H), 6.50 (d, J = 2.3 Hz, 1H), 6.86 (d, J = 2.0 Hz, 1H), 6.95 (dd, J = 8.4, 2.4 Hz, 1H), 7.62 (d, J = 8.7 Hz, 1H), 8.38 (m, 4H, H-1′).

^{13}C NMR (101 MHz, $CDCl_3$) δ (ppm): 39.4, 55.4, 66.6, 77.8, 96.9, 106.5, 110.6, 114.9, 118.4, 118.6, 123.6, 124.7, 131.3, 132.0, 134.6, 150.8, 151.3, 156.3, 160.4, 161.1, 163.0.

3.- NMR spectra and HPLC chromatogram

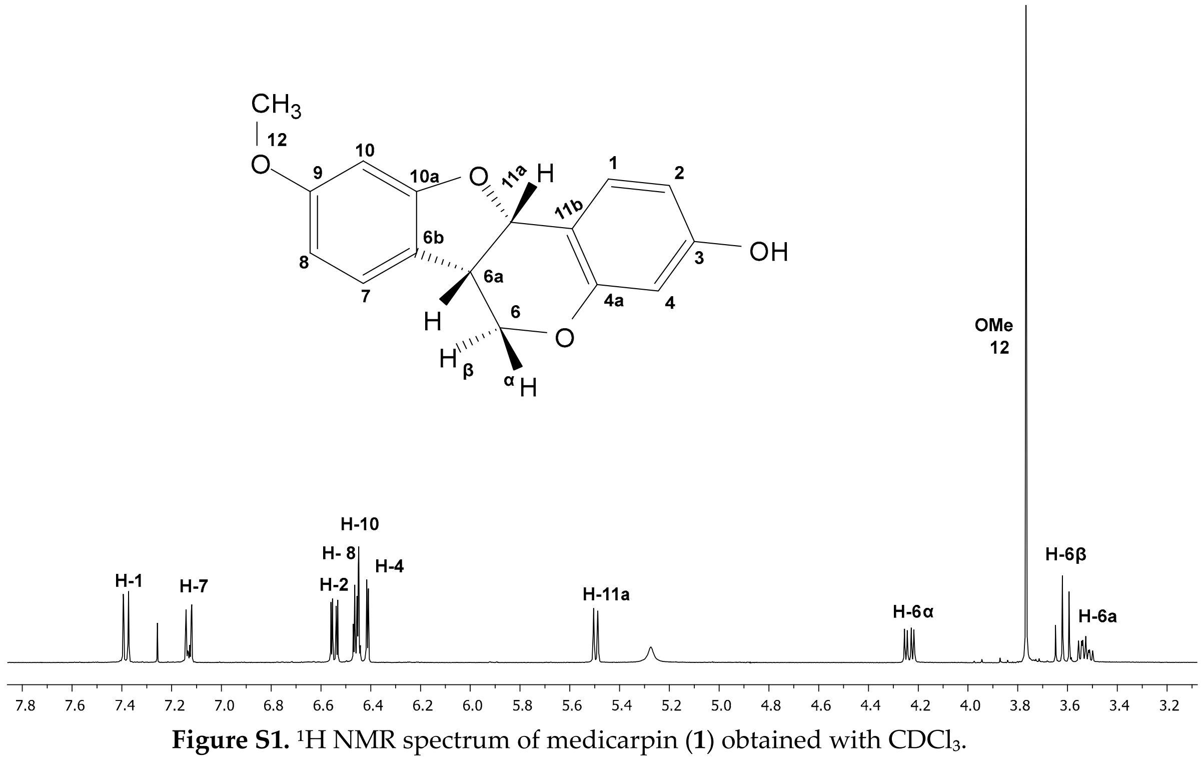

Figure S1. ^{1}H NMR spectrum of medicarpin (**1**) obtained with $CDCl_3$.

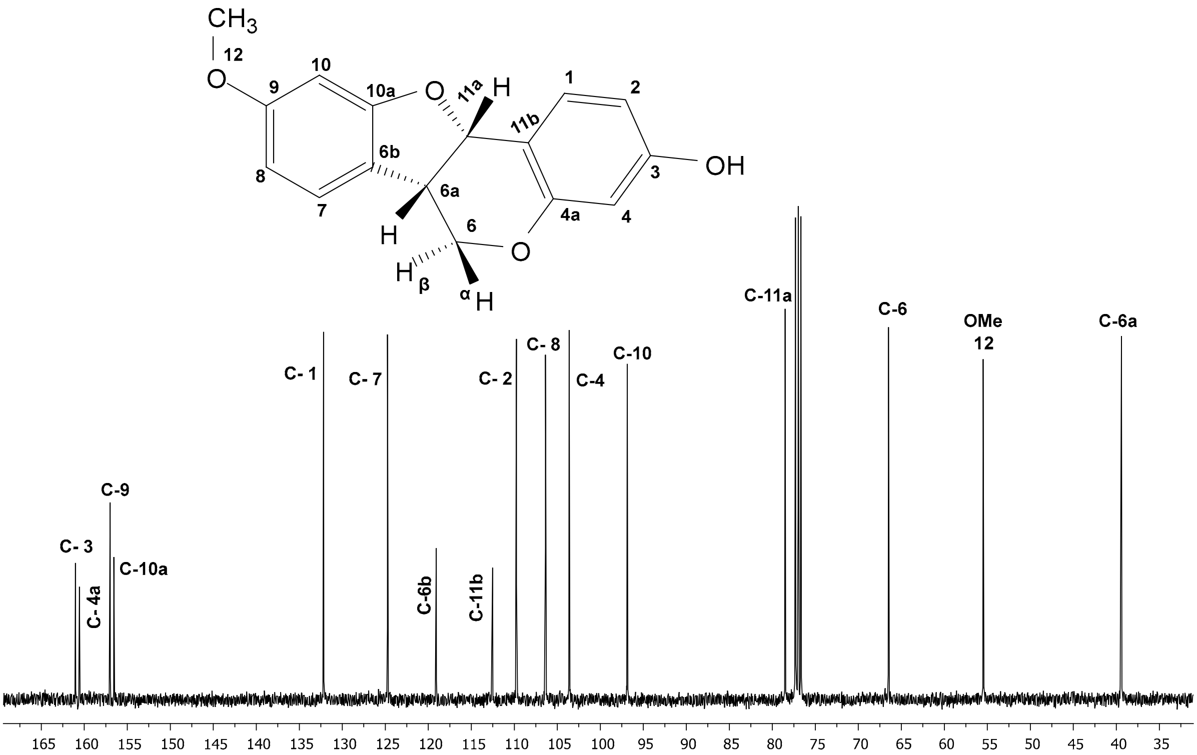

Figure S2. ^{13}C NMR spectrum of medicarpin (**1**) obtained with $CDCl_3$.

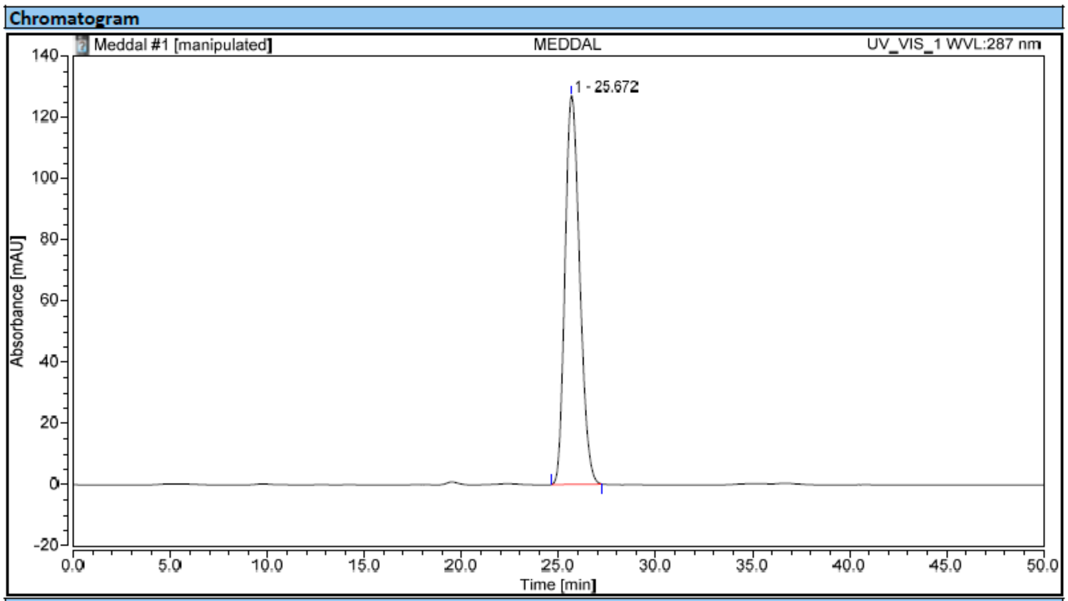

Integration Results

No.	Peak Name	Retention Time min	Area mAU*min	Height mAU	Relative Area %	Relative Height %	Amount n.a.
1		25.672	111.497	127.034	100.00	100.00	n.a.
Total:			**111.497**	**127.034**	**100.00**	**100.00**	

Figure S3. HPLC chromatogram of medicarpin (**1**).

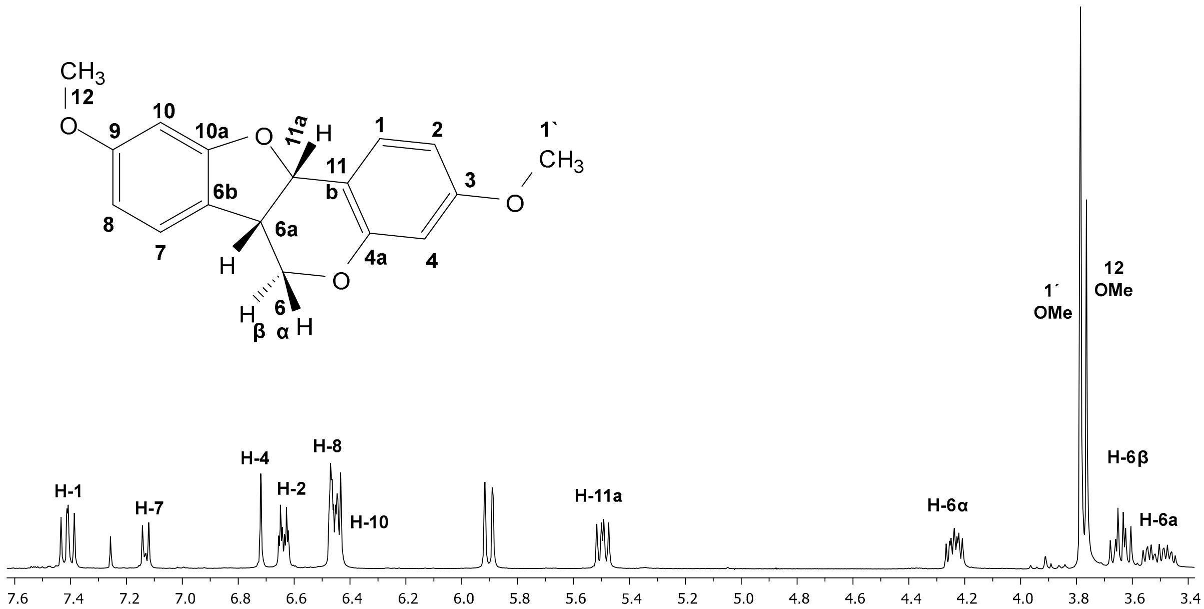

Figure S4. ^{1}H NMR spectrum of methyl ether derivative of medicarpin (**2**) obtained with $CDCl_3$.

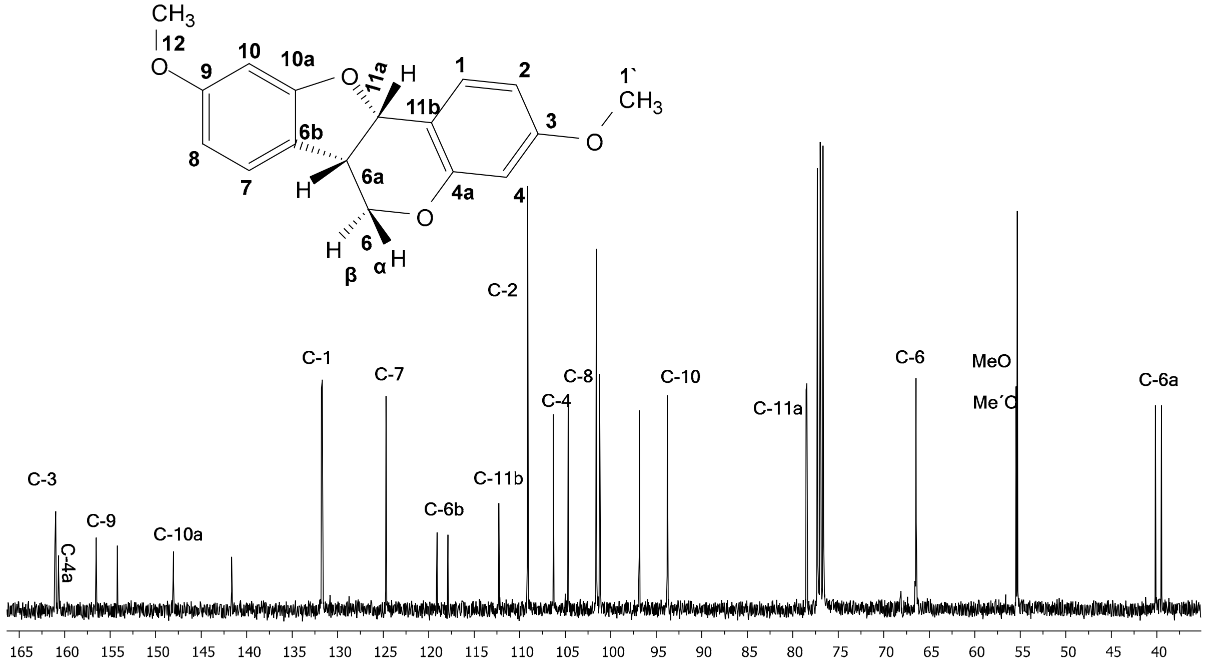

Figure S5. ^{13}C NMR spectrum of methyl ether derivative of medicarpin (**2**) obtained with $CDCl_3$.

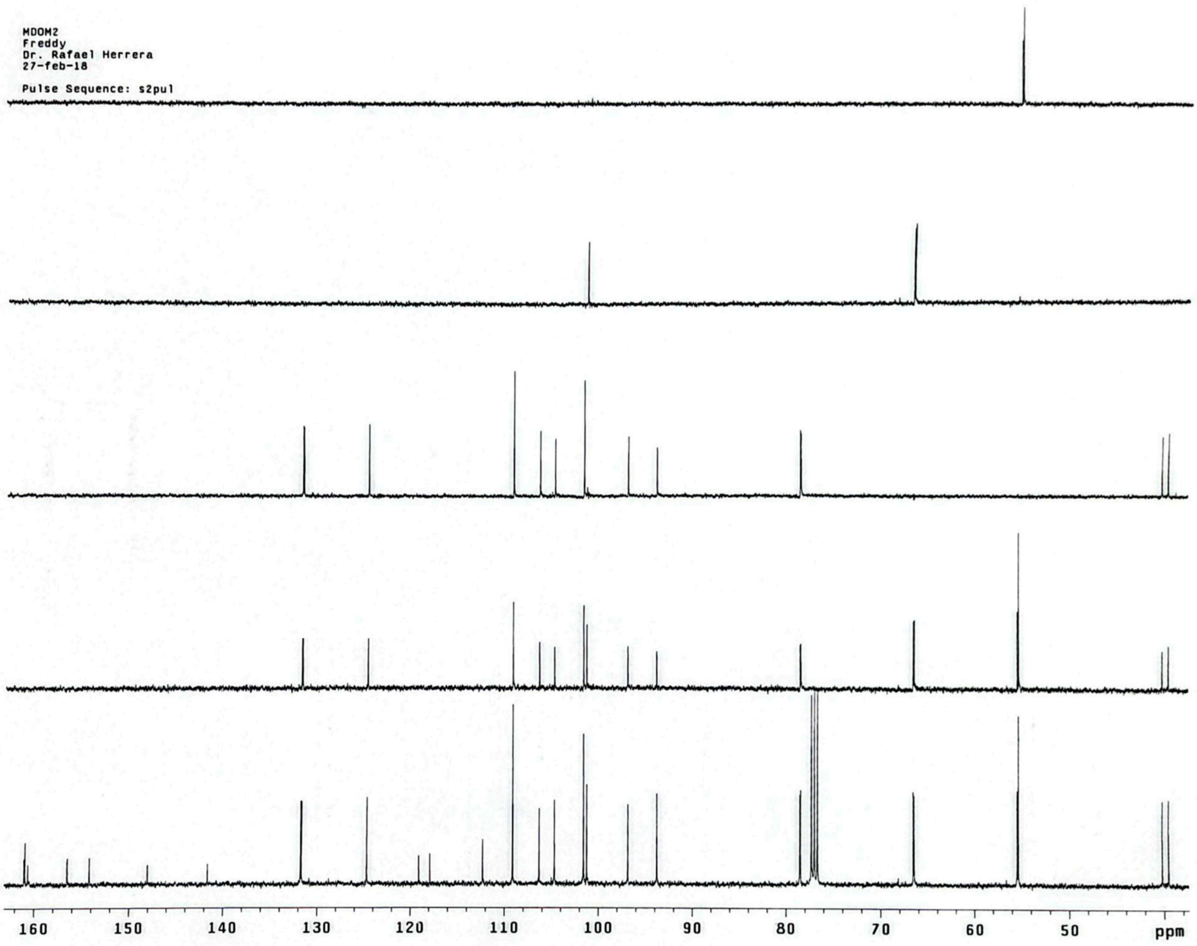

Figure S6. 2D DEPT spectrum of methyl ether derivative of medicarpin (**2**) obtained with $CDCl_3$.

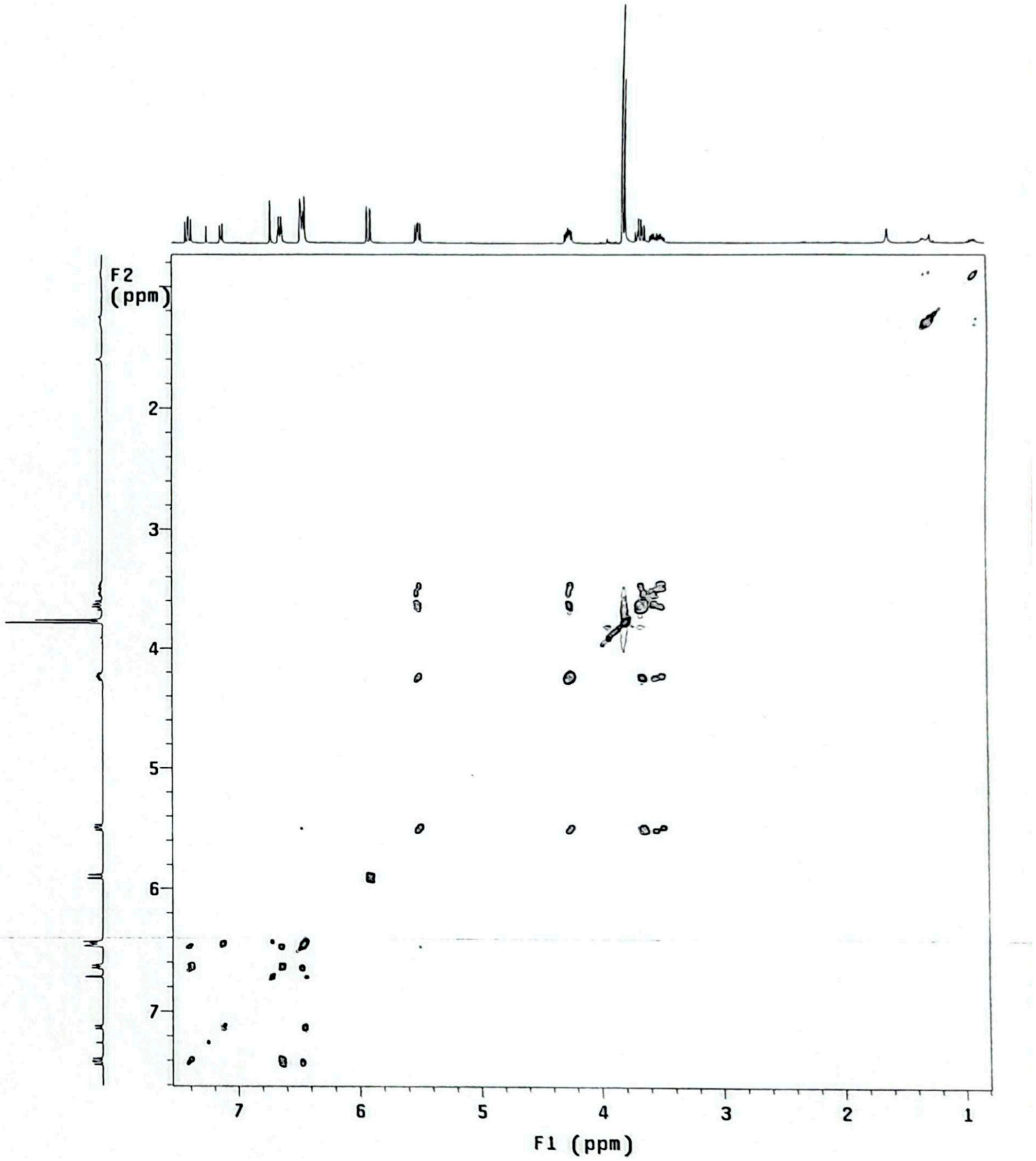

Figure S7. 2D TOCSY spectrum of methyl ether derivative of medicarpin (**2**) obtained with $CDCl_3$.

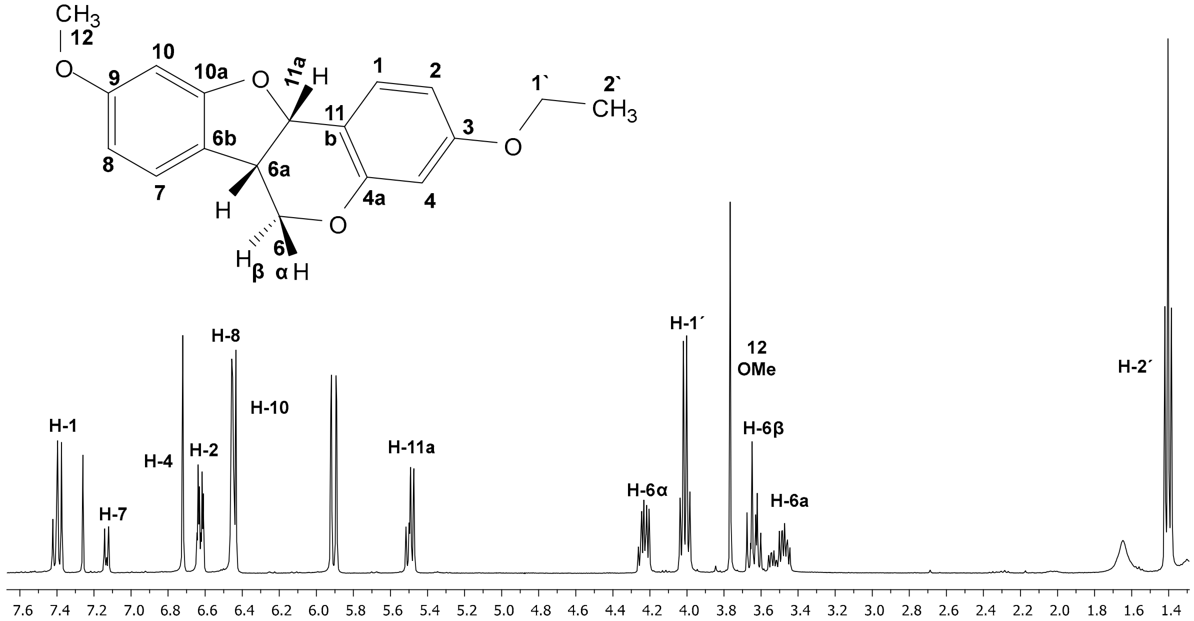

Figure S8. ^{1}H NMR spectrum of ethyl ether derivative of medicarpin (**3**) obtained with $CDCl_3$.

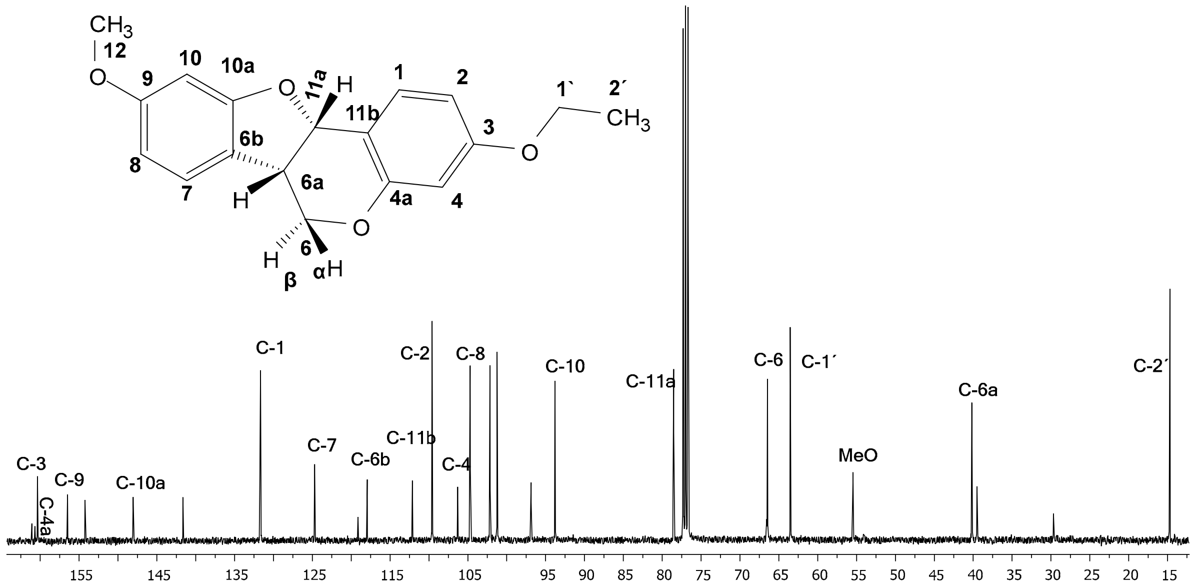

Figure S9. ^{13}C NMR spectrum of ethyl ether derivative of medicarpin (**3**) obtained with $CDCl_3$.

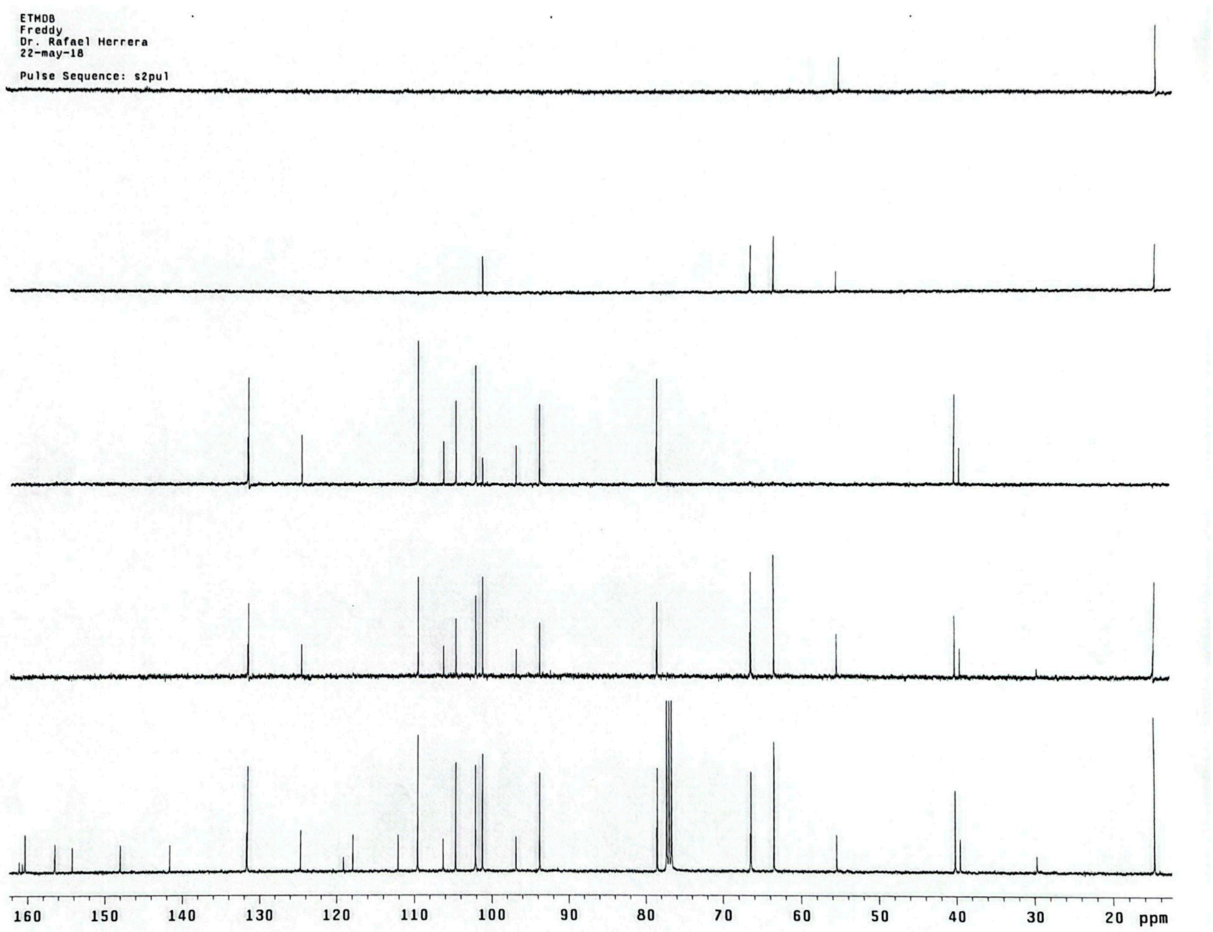

Figure S10. 2D DEPT spectrum of ethyl ether derivative of medicarpin (**3**) obtained with $CDCl_3$.

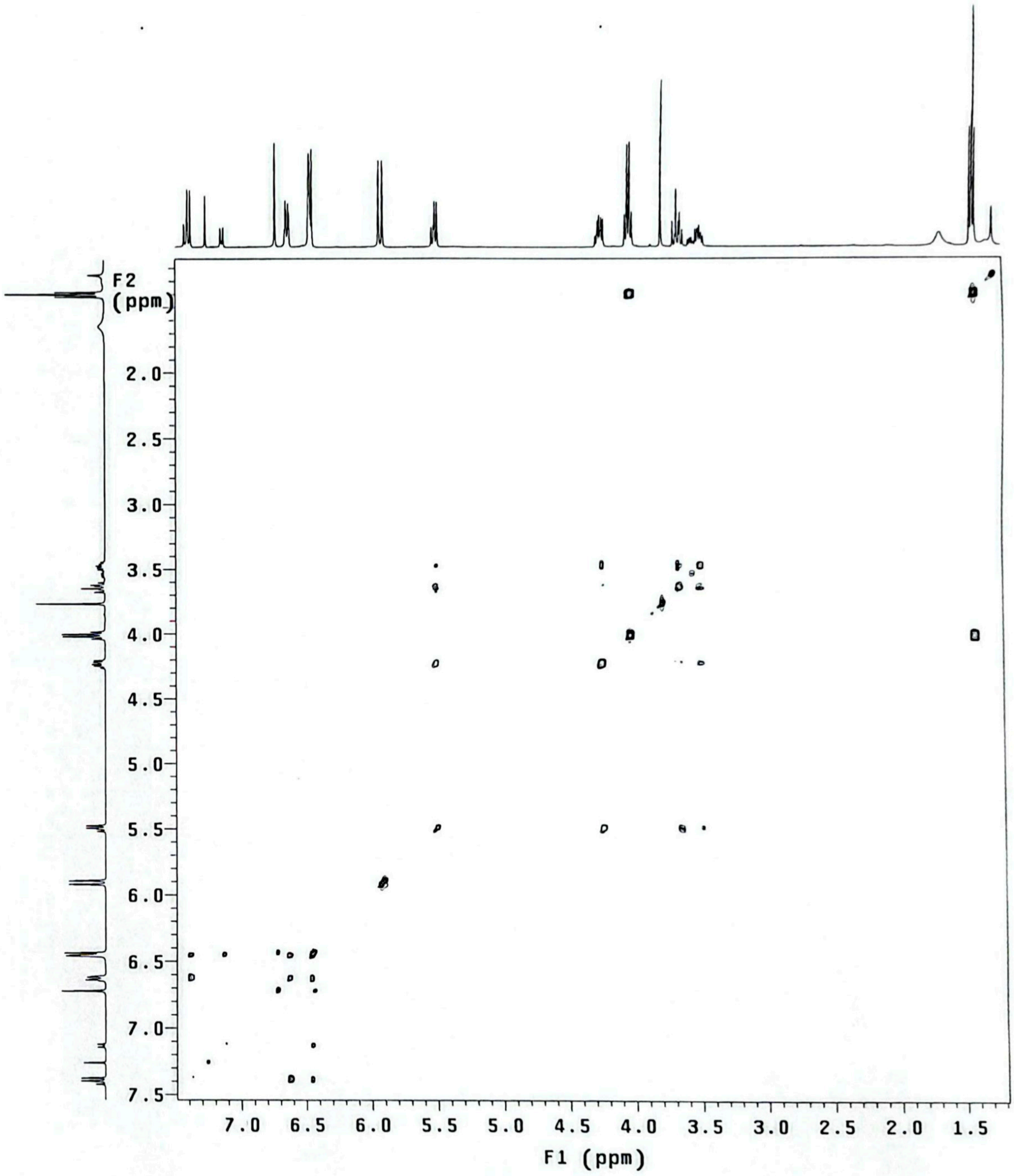

Figure S11. 2D TOCSY spectrum of ethyl ether derivative of medicarpin (**3**) obtained with $CDCl_3$.

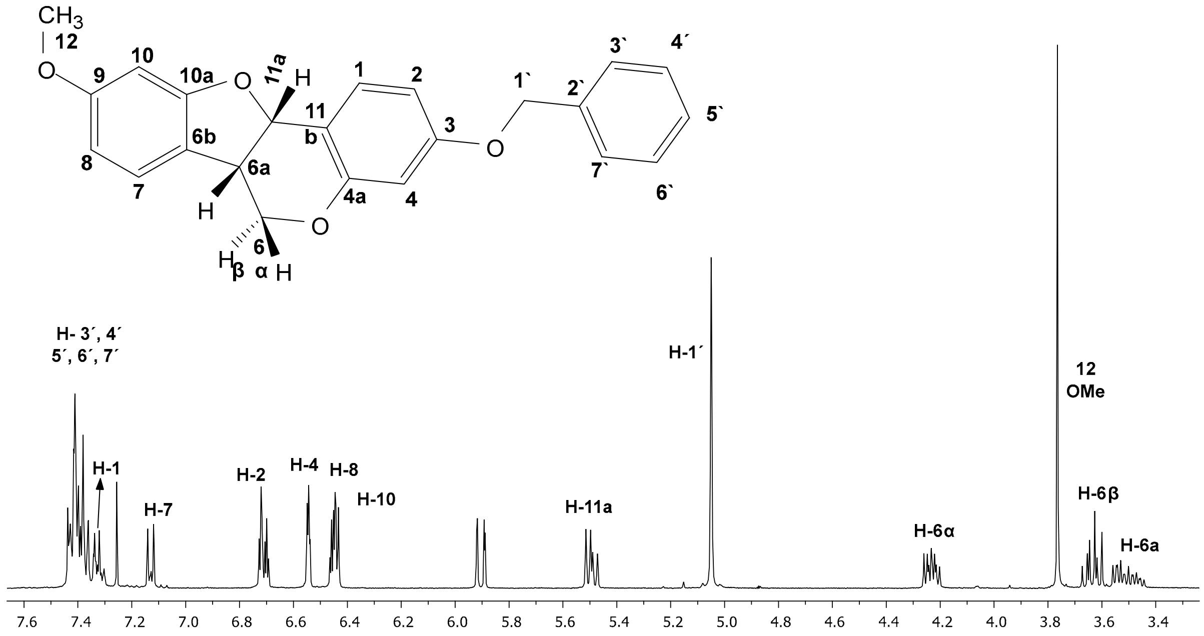

Figure S12. ^{1}H NMR spectrum of benzyl ether derivative of medicarpin (**4**) obtained with $CDCl_3$.

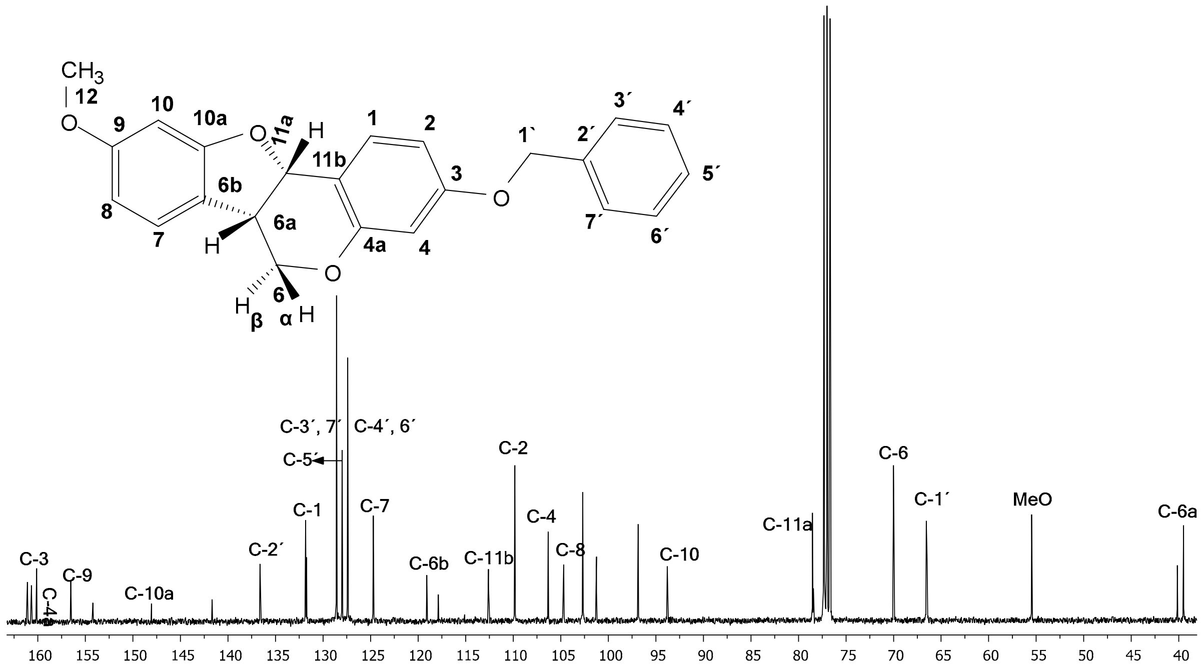

Figure S13. ^{13}C NMR spectrum of benzyl ether derivative of medicarpin (**4**) obtained with $CDCl_3$.

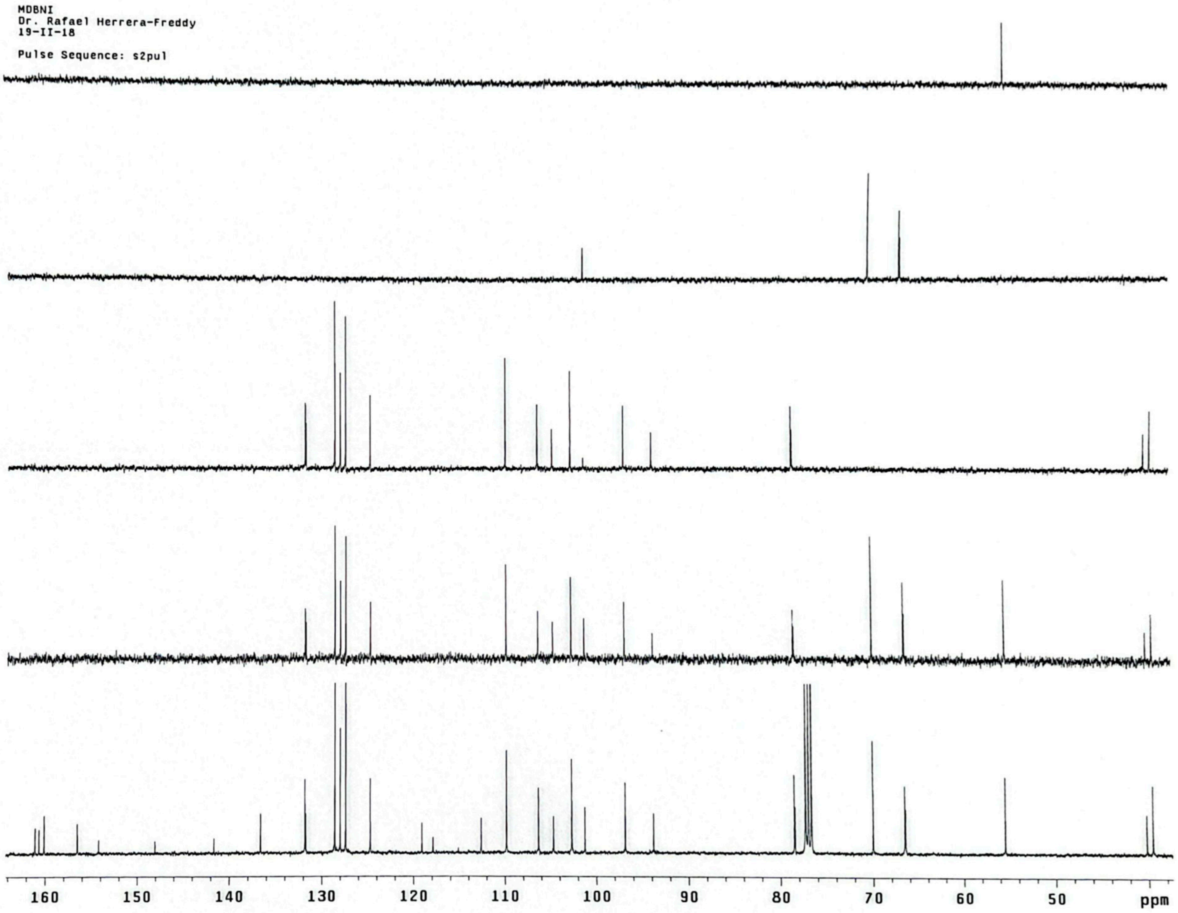

Figure S14. 2D DEPT spectrum of benzyl ether derivative of medicarpin (**4**) obtained with $CDCl_3$.

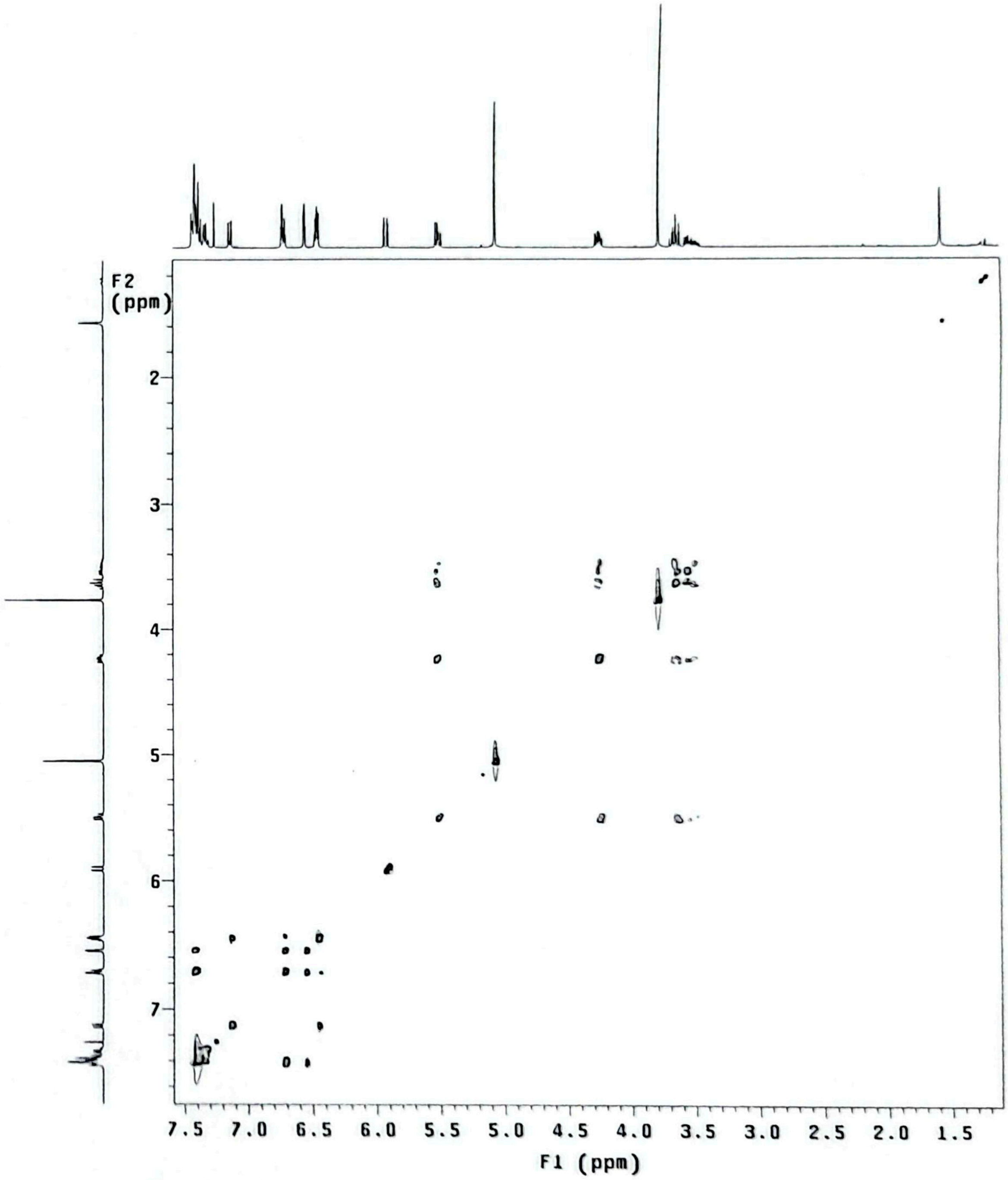

Figure S15. 2D TOCSY spectrum of benzyl ether derivative of medicarpin (**4**) obtained with $CDCl_3$.

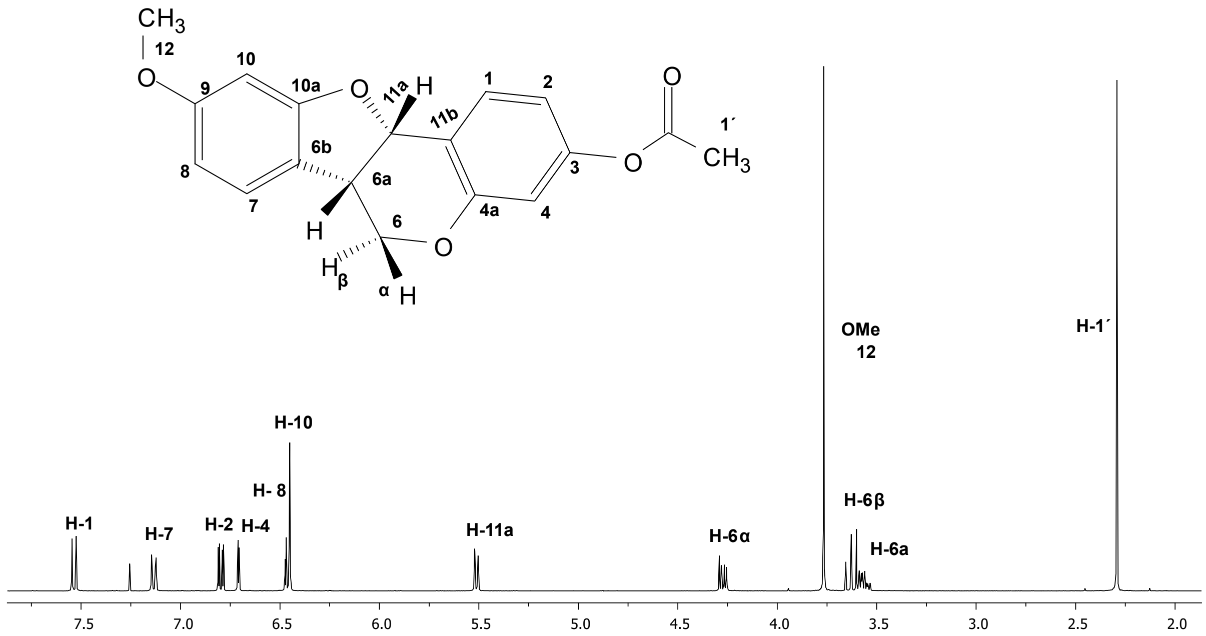

Figure S16. ^{1}H NMR spectrum of acetylated derivative of medicarpin (**5**) obtained with $CDCl_3$.

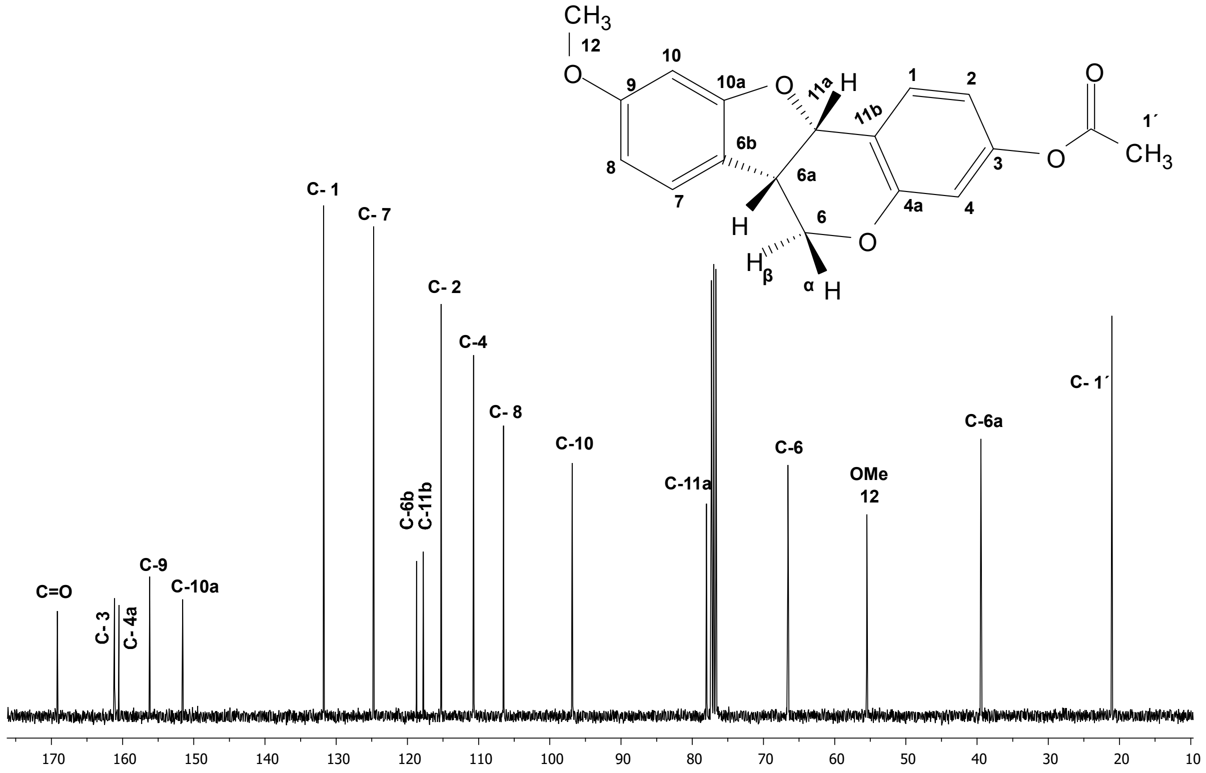

Figure S17. ^{13}C NMR spectrum of acetylated derivative of medicarpin (**5**) obtained with $CDCl_3$.

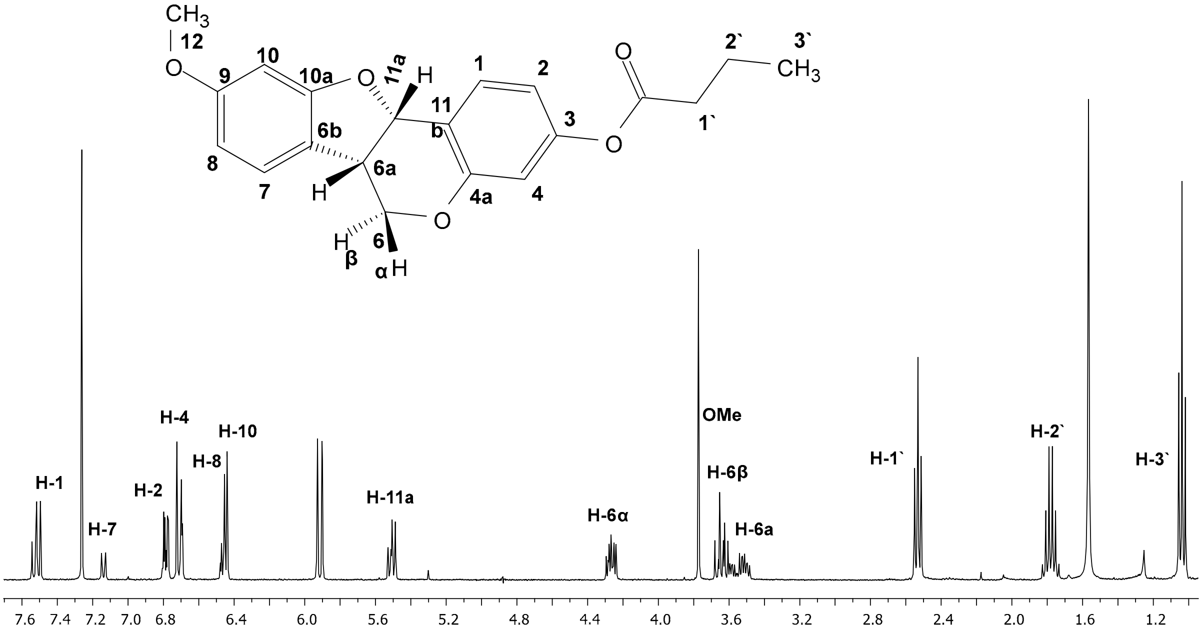

Figure S18. ^{1}H NMR spectrum of butyrate derivative of medicarpin (**6**) obtained with $CDCl_3$.

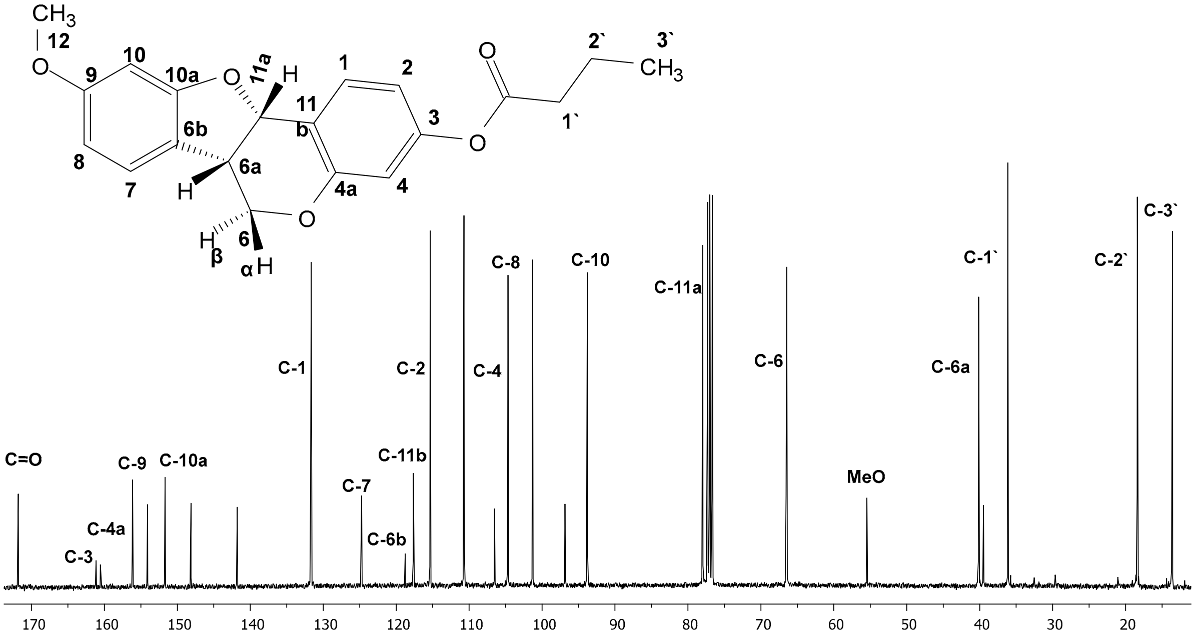

Figure S19. ^{13}C NMR spectrum of butyrate derivative of medicarpin (**6**) obtained with $CDCl_3$.

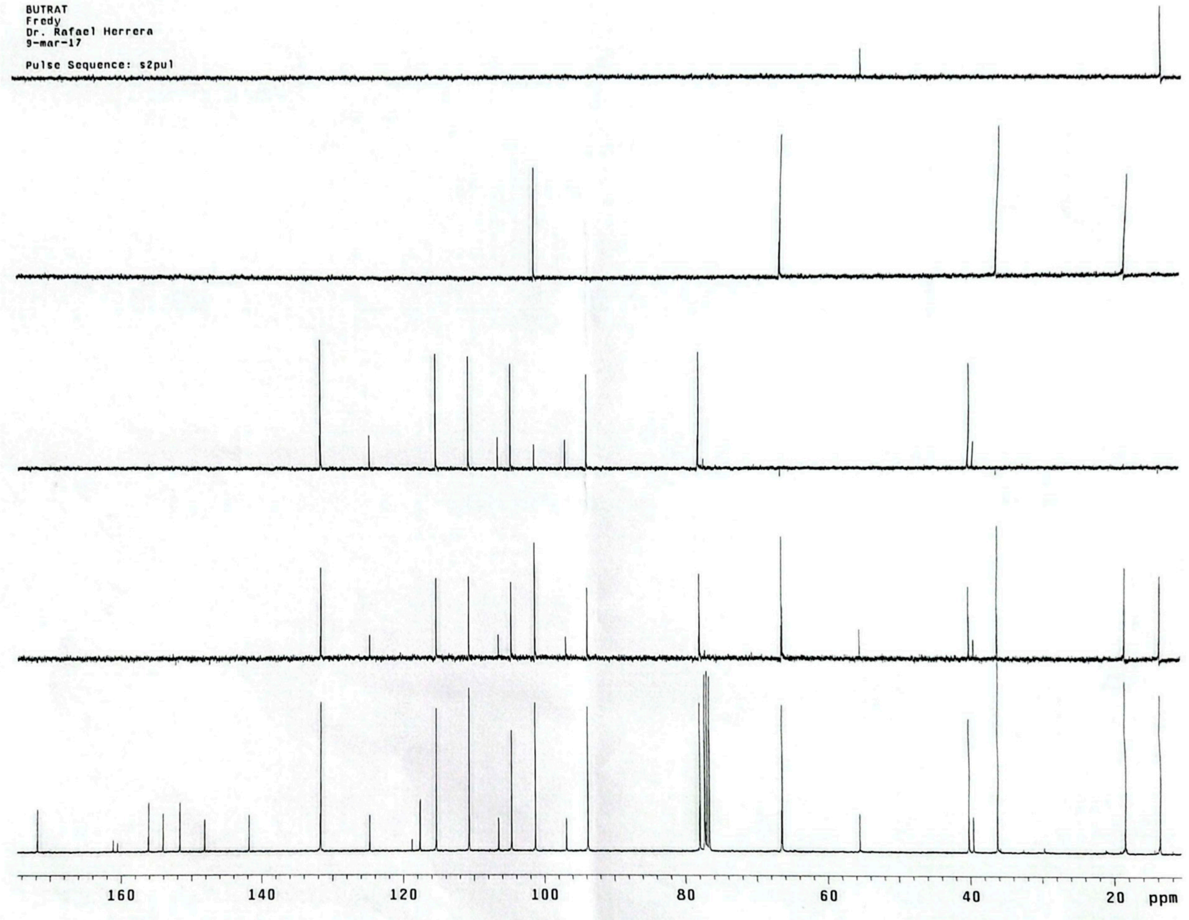

Figure S20. 2D DEPT spectrum of butyrate derivative of medicarpin (**6**) obtained with $CDCl_3$.

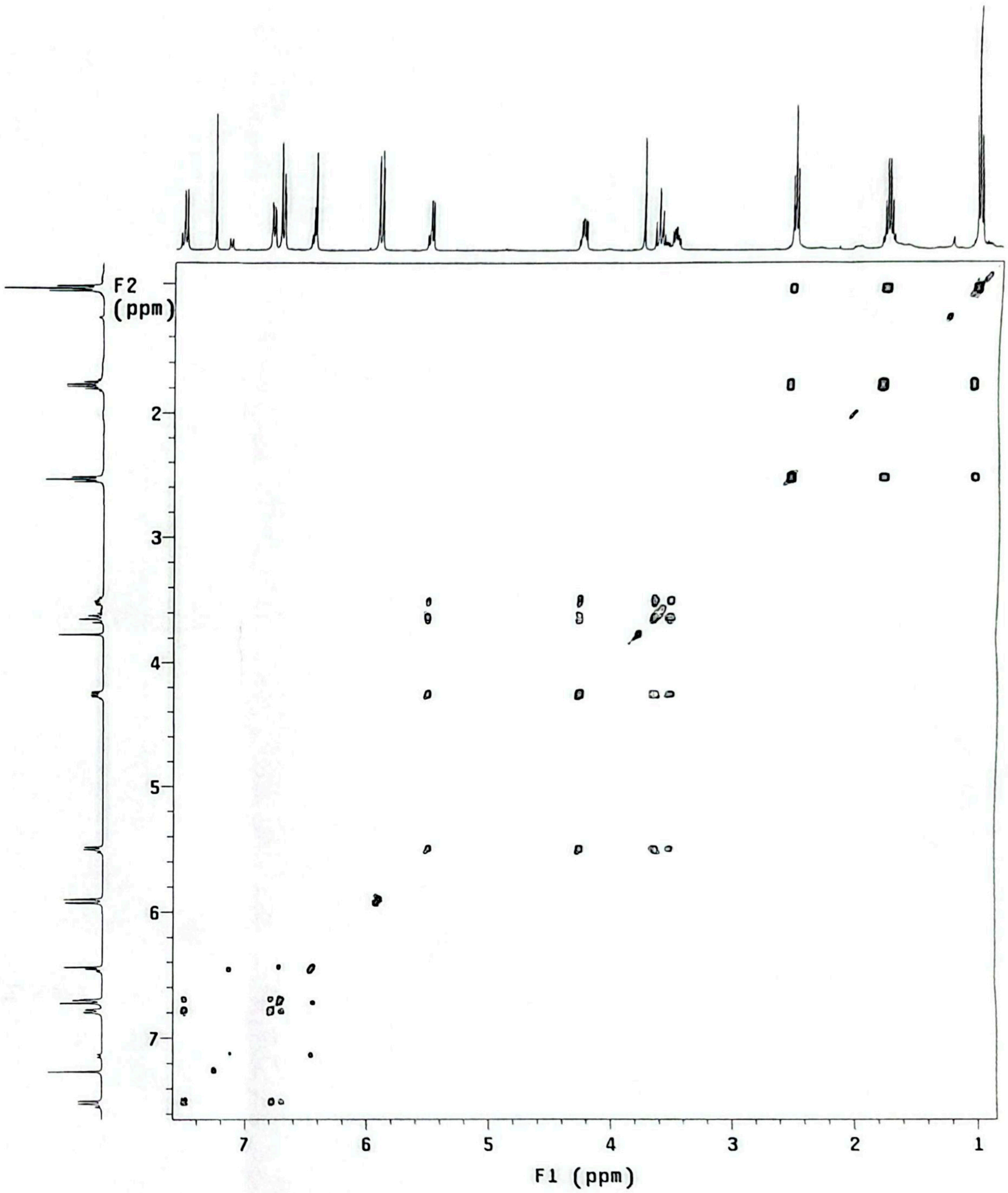

Figure S21. 2D TOCSY spectrum of butyrate derivative of medicarpin (**6**) obtained with $CDCl_3$.

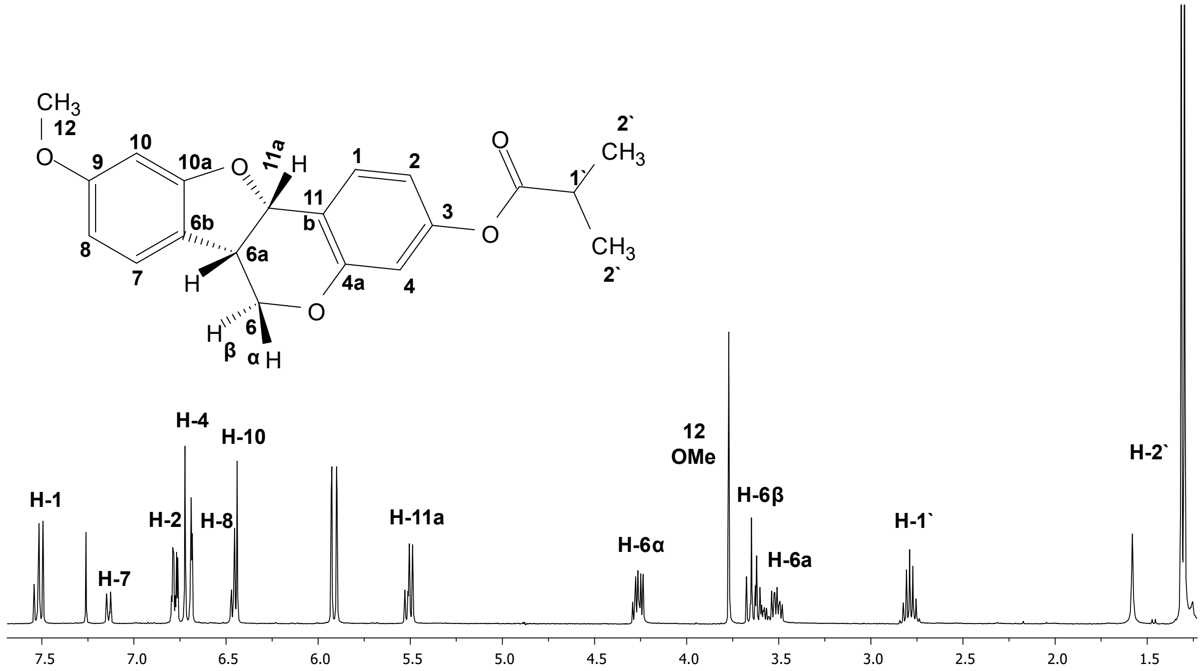

Figure S22. ^{1}H NMR spectrum of isobutyrate derivative of medicarpin (**7**) obtained with $CDCl_3$.

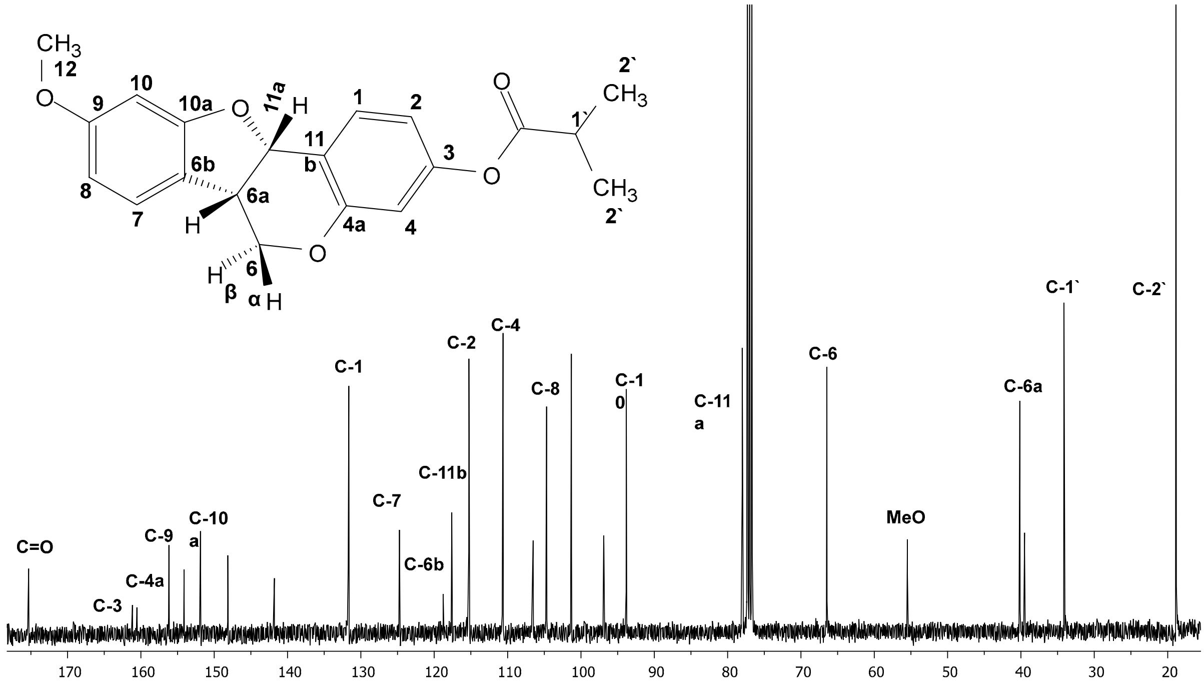

Figure S23. ^{13}C NMR spectrum of isobutyrate derivative of medicarpin (**7**) obtained with $CDCl_3$.

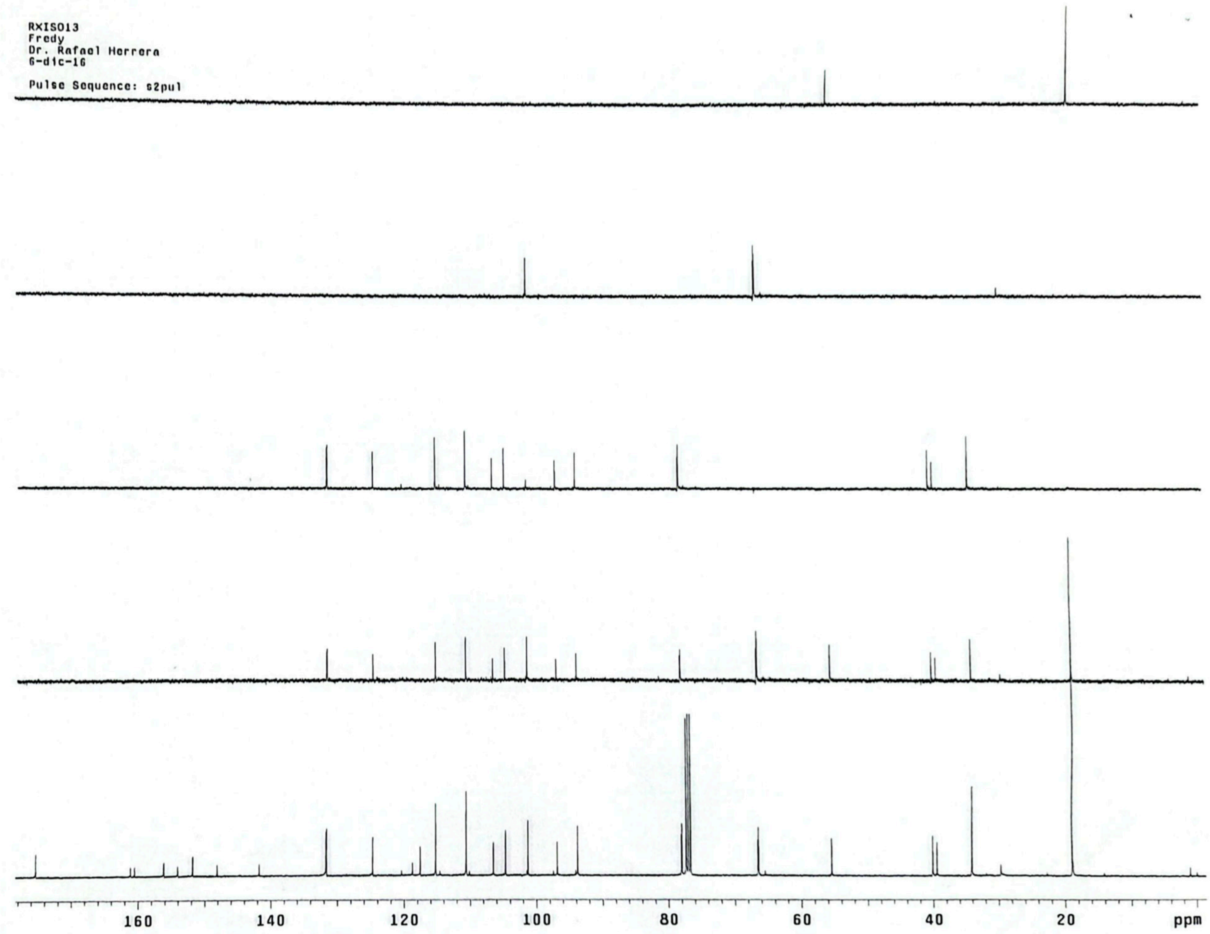

Figure S24. 2D DEPT spectrum of isobutyrate derivative of medicarpin (**7**) obtained with $CDCl_3$.

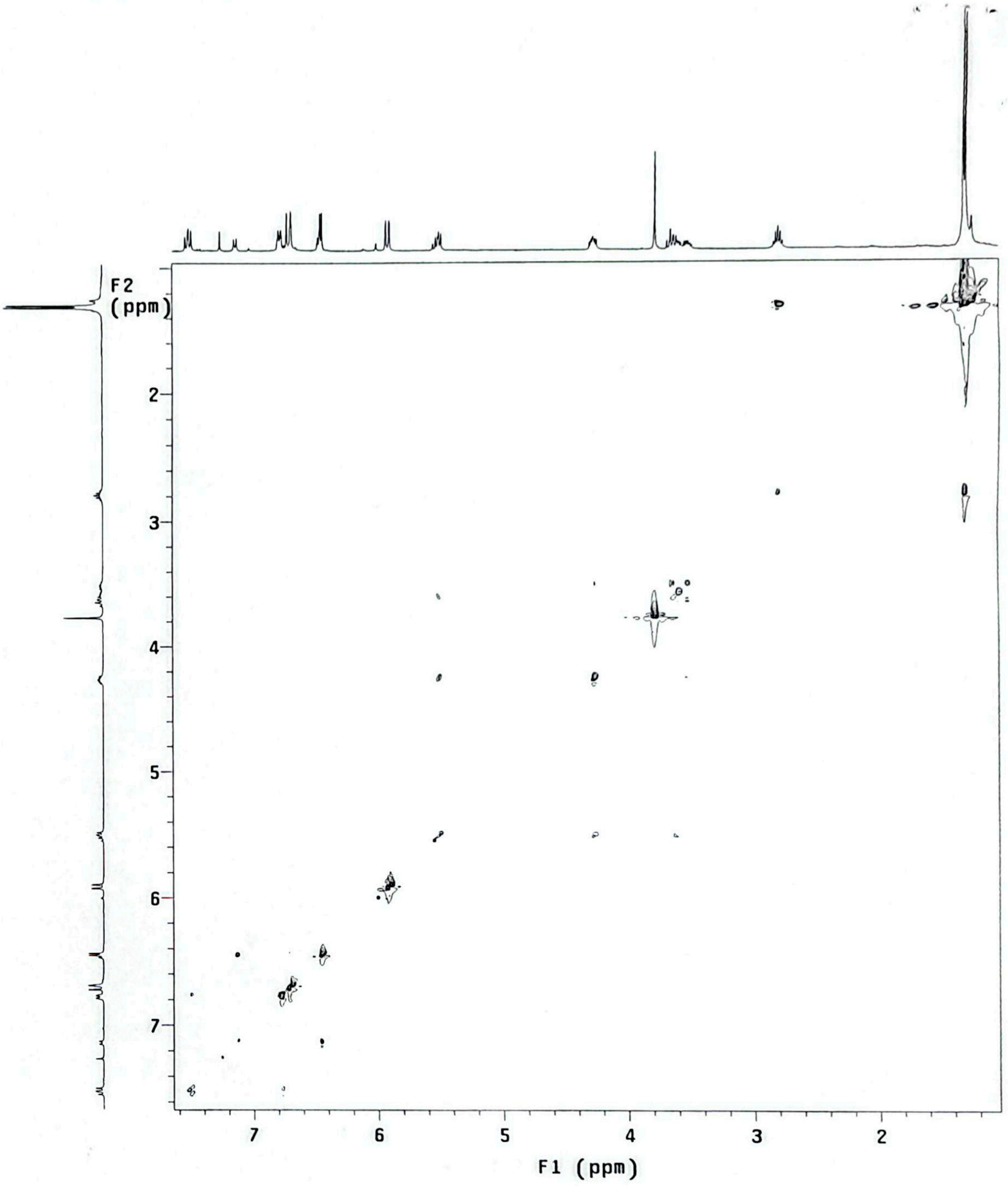

Figure S25. 2D TOCSY spectrum of isobutyrate derivative of medicarpin (**7**) obtained with $CDCl_3$.

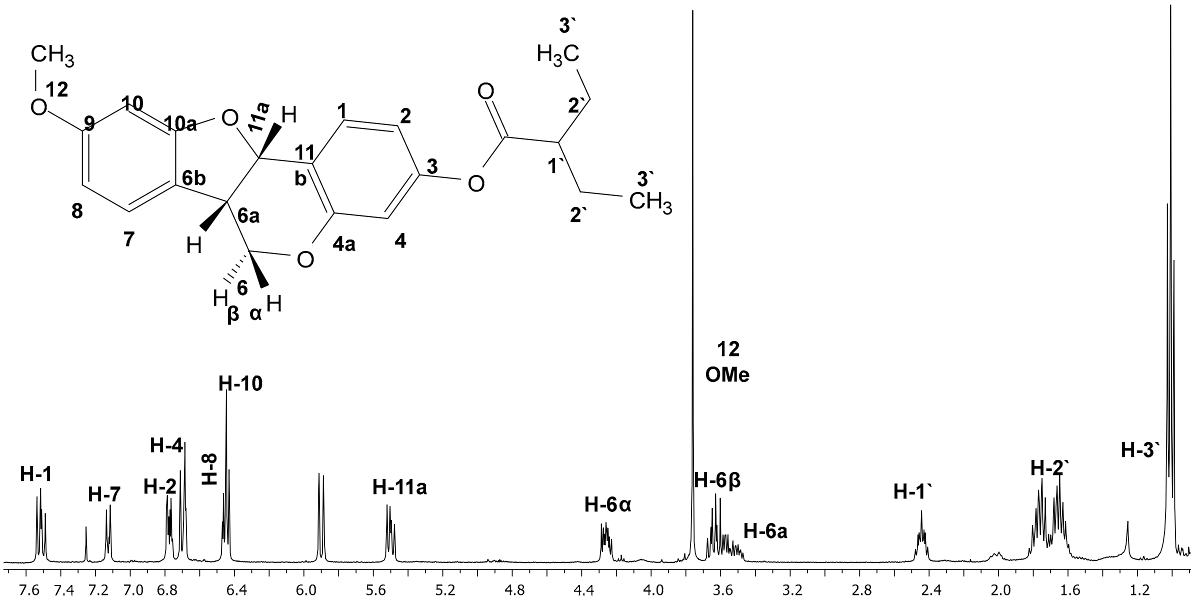

Figure S26. ^{1}H NMR spectrum of 2-ethylbutanoate derivative of medicarpin (**8**) obtained with $CDCl_3$.

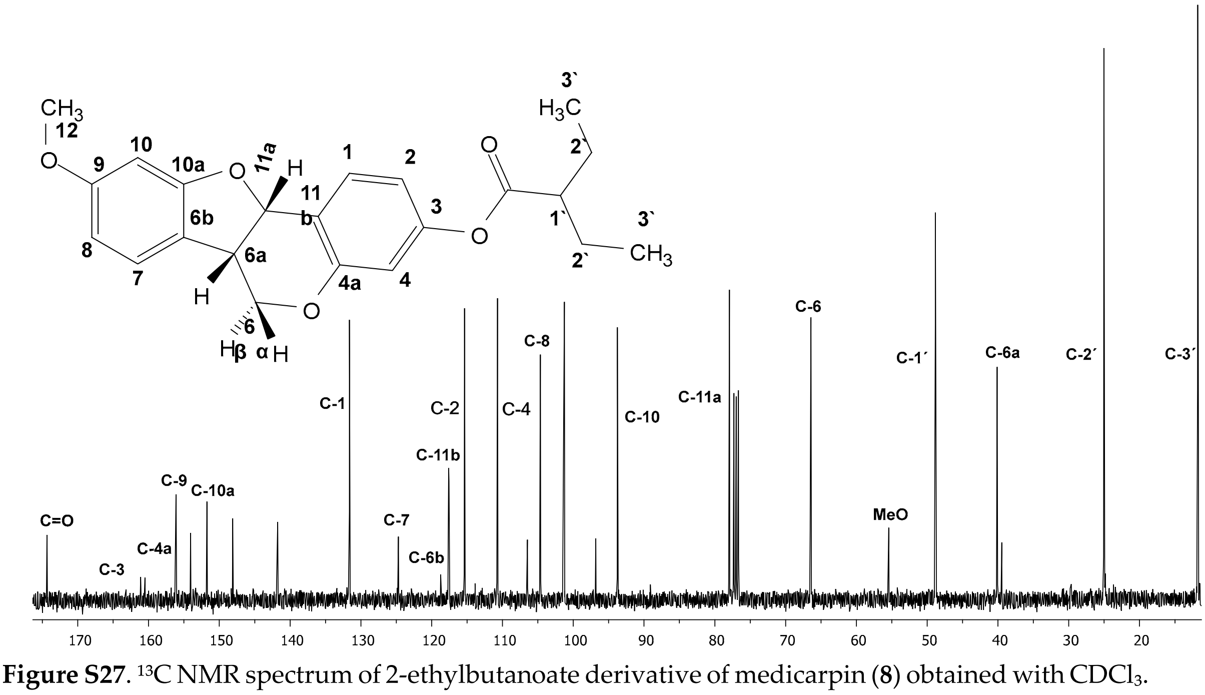

Figure S27. ^{13}C NMR spectrum of 2-ethylbutanoate derivative of medicarpin (**8**) obtained with $CDCl_3$.

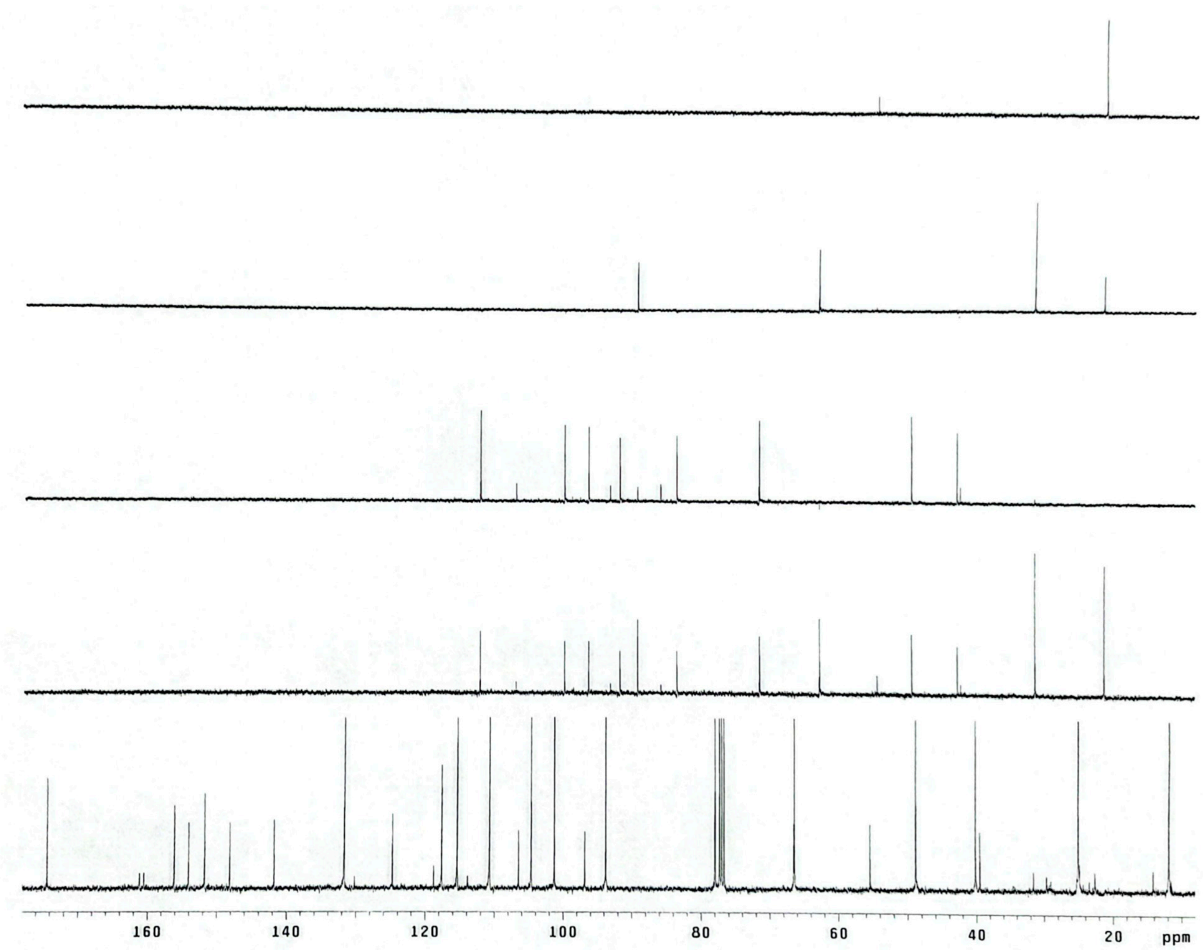

Figure S28. 2D DEPT spectrum of 2-ethylbutanoate derivative of medicarpin (**8**) obtained with $CDCl_3$.

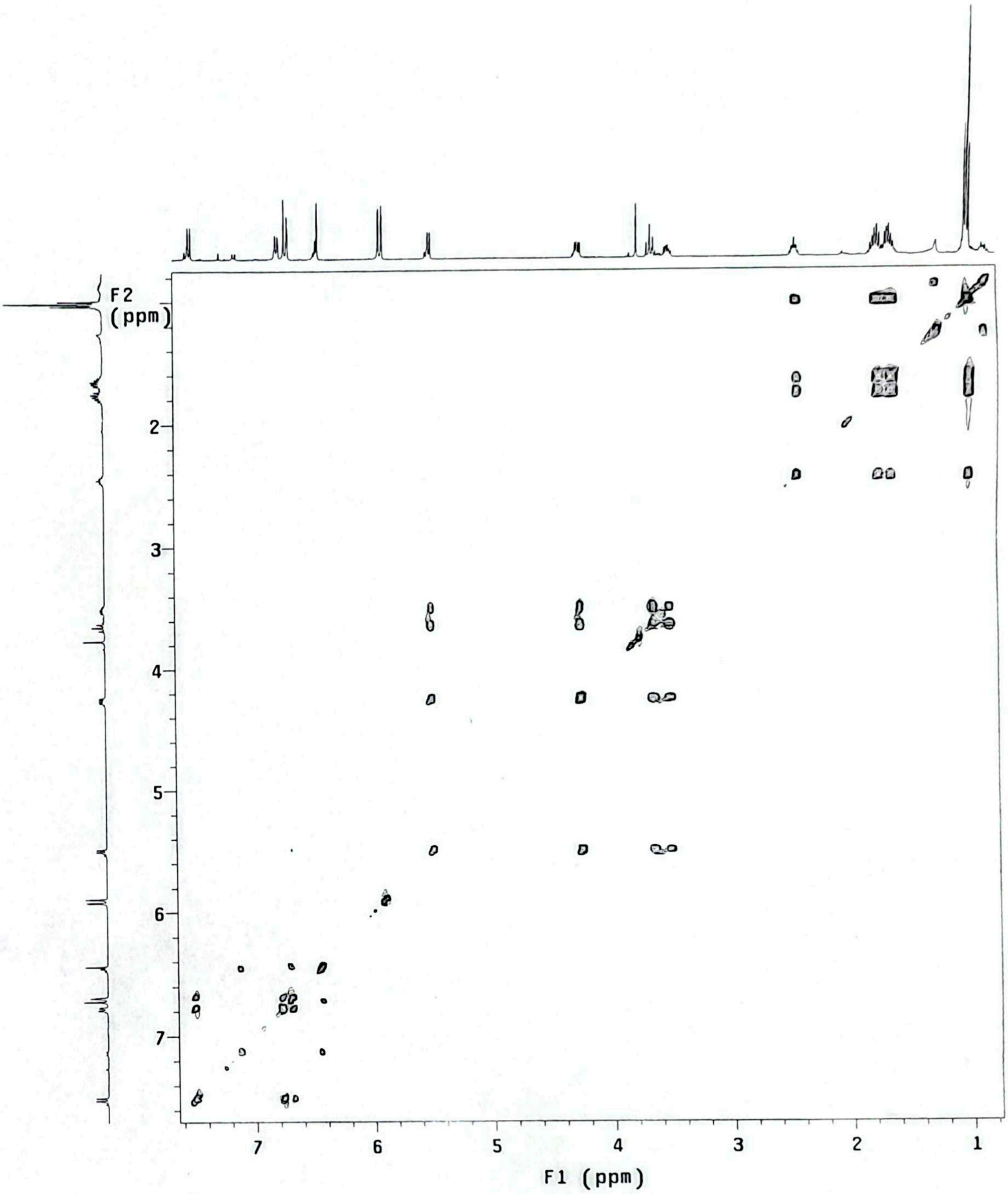

Figure S29. 2D TOCSY spectrum of 2-ethylbutanoate derivative of medicarpin (**8**) obtained with $CDCl_3$.

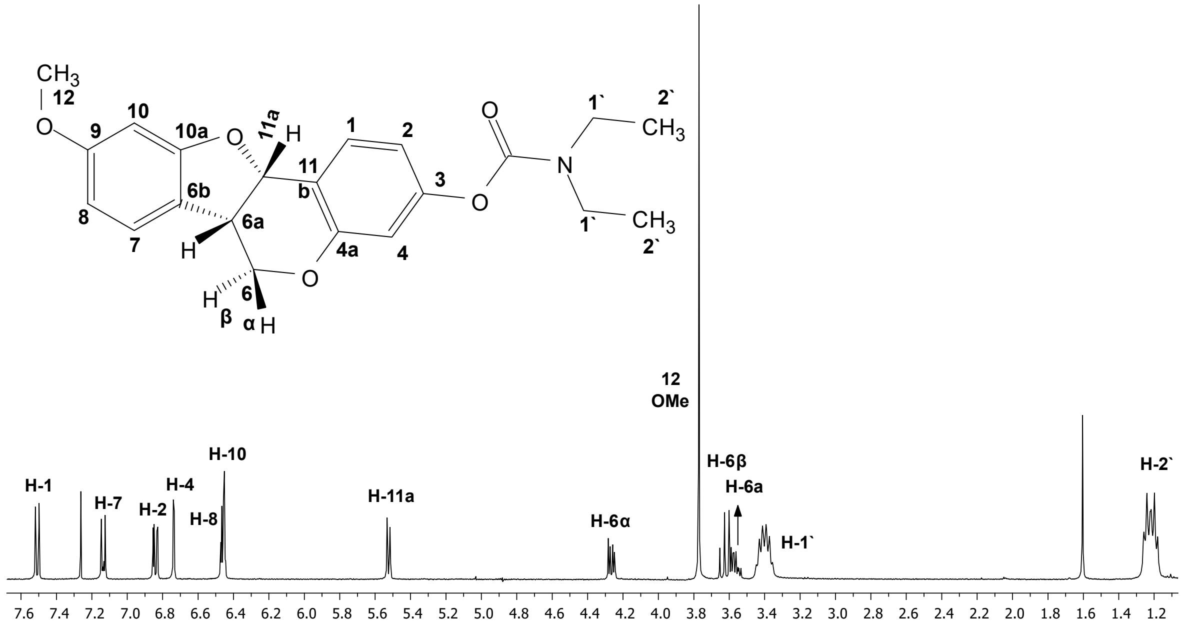

Figure S30. ^{1}H NMR spectrum of diethylcarbamate derivative of medicarpin (**9**) obtained with $CDCl_3$.

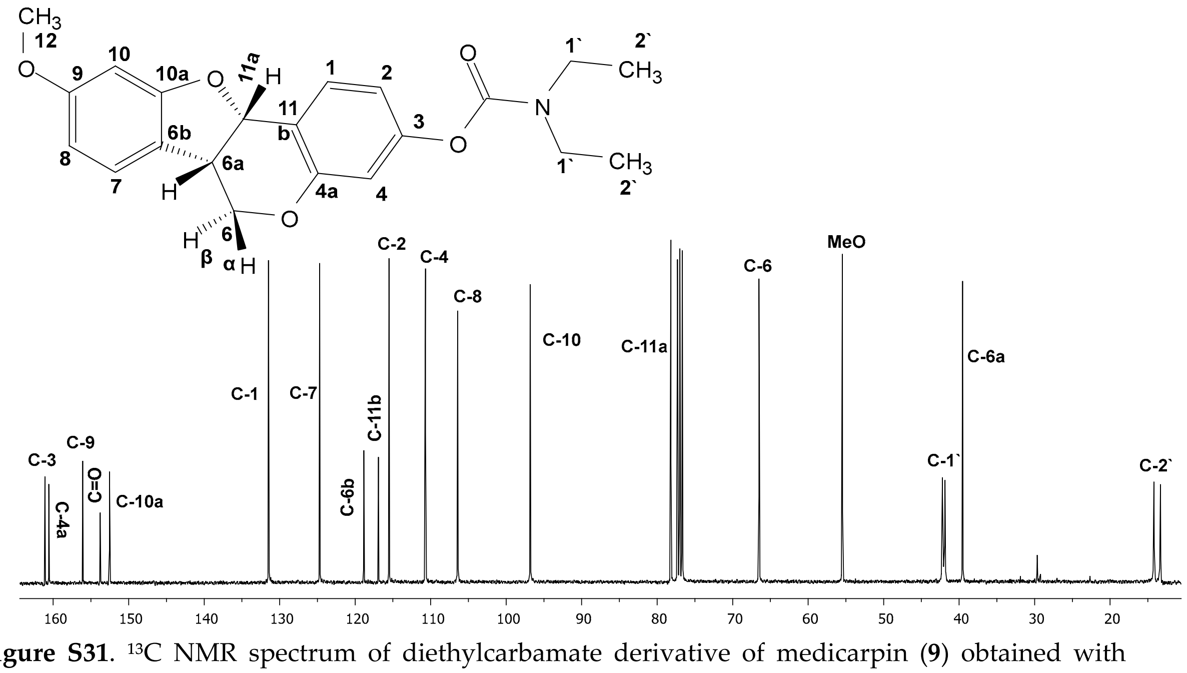

Figure S31. ^{13}C NMR spectrum of diethylcarbamate derivative of medicarpin (**9**) obtained with $CDCl_3$.

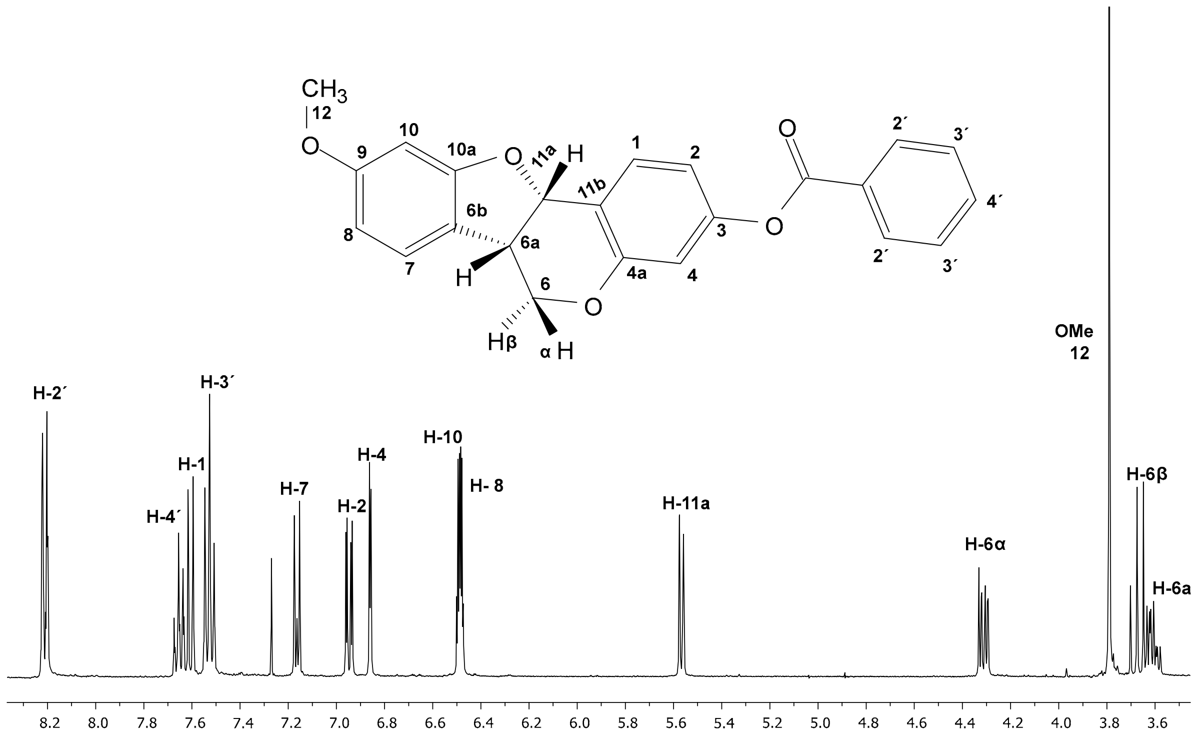

Figure S32. ^{1}H NMR spectrum of benzoate derivative of medicarpin (**10**) obtained with $CDCl_3$.

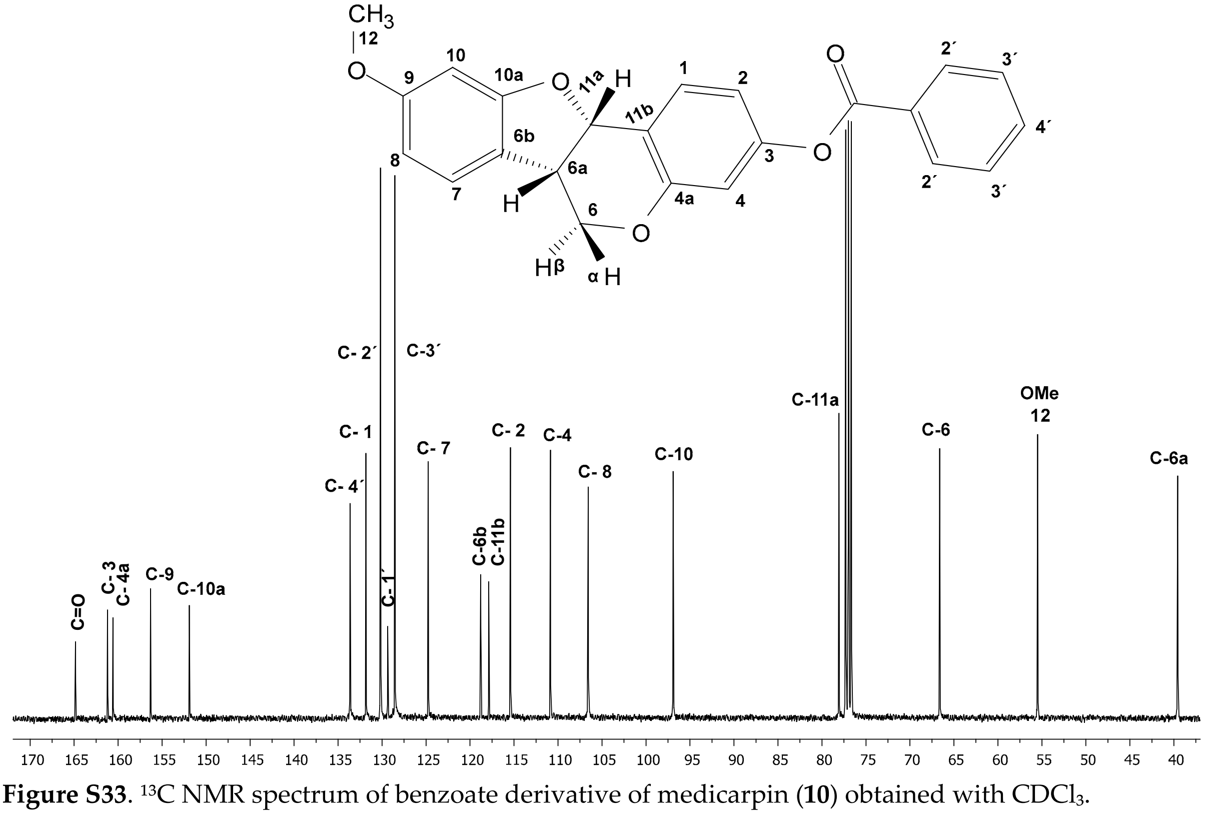

Figure S33. ^{13}C NMR spectrum of benzoate derivative of medicarpin (**10**) obtained with $CDCl_3$.

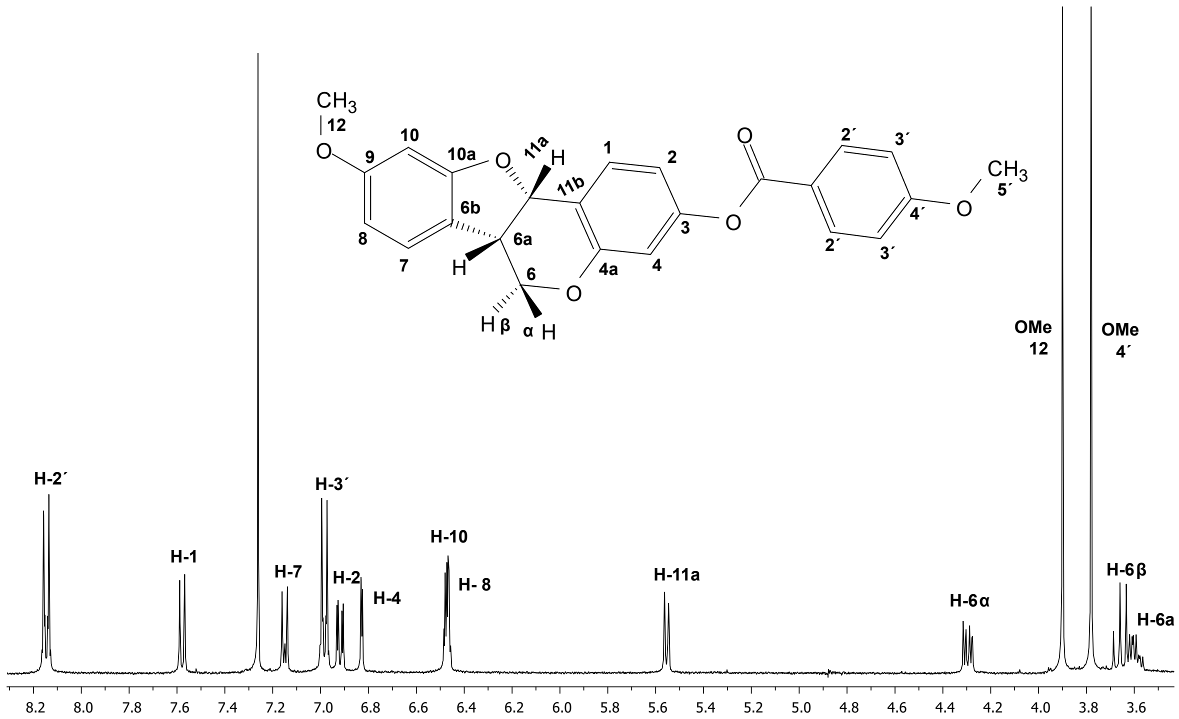

Figure S34. ^{1}H NMR spectrum of 4-methoxybenzoate derivative of medicarpin (**11**) obtained with $CDCl_3$.

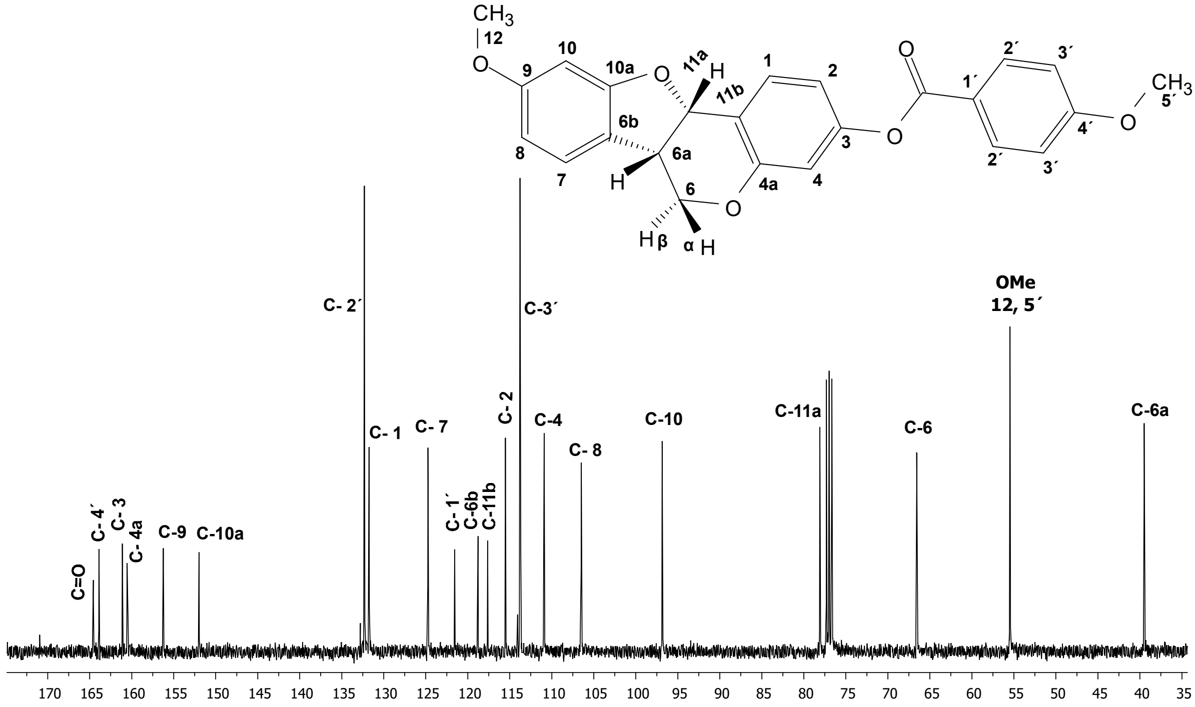

Figure S35. ^{13}C NMR spectrum of 4-methoxybenzoate derivative of medicarpin (**11**) obtained with $CDCl_3$.

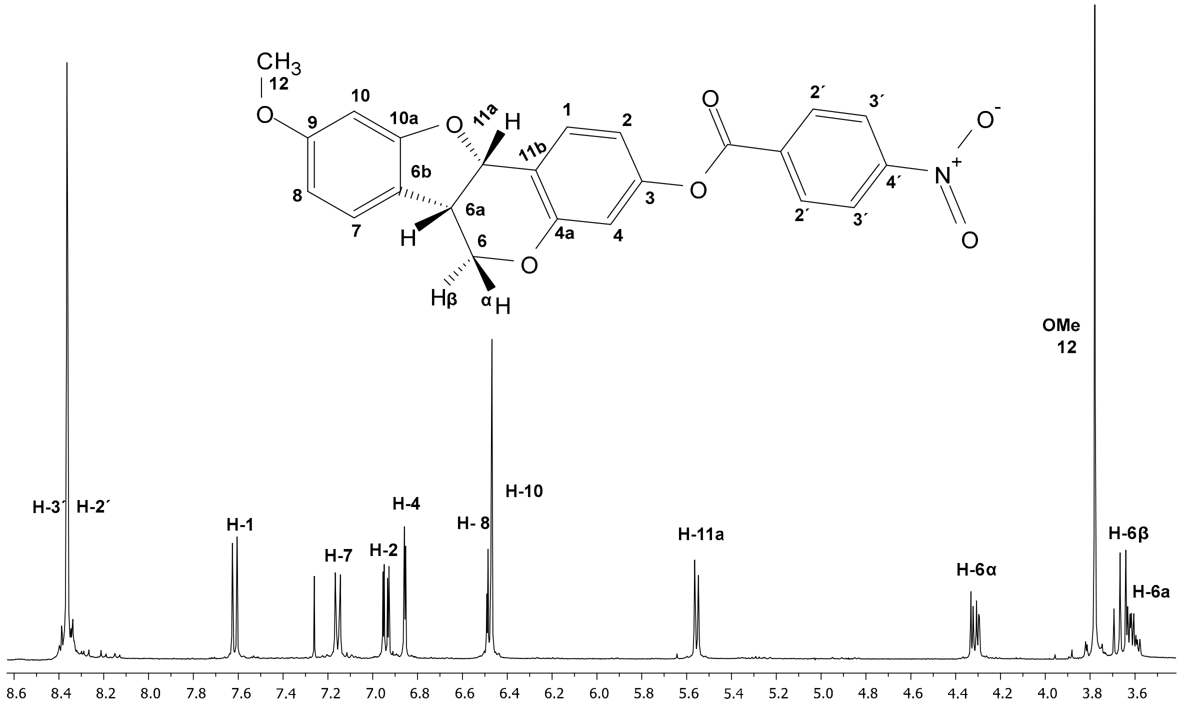

Figure S36. ^{1}H NMR spectrum of 4-nitrobenzoate derivative of medicarpin (**12**) obtained with $CDCl_3$.

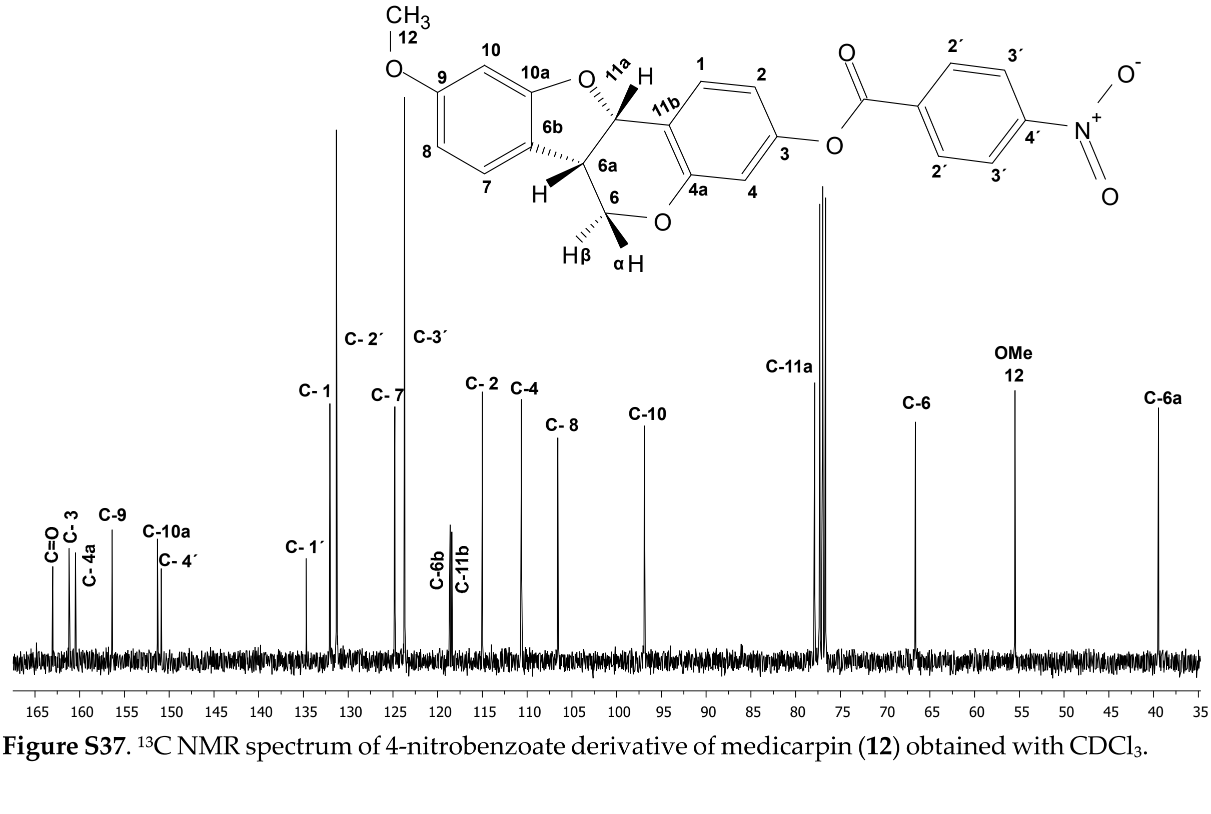

Figure S37. ^{13}C NMR spectrum of 4-nitrobenzoate derivative of medicarpin (**12**) obtained with $CDCl_3$.

4.- RMSD molecular dynamics

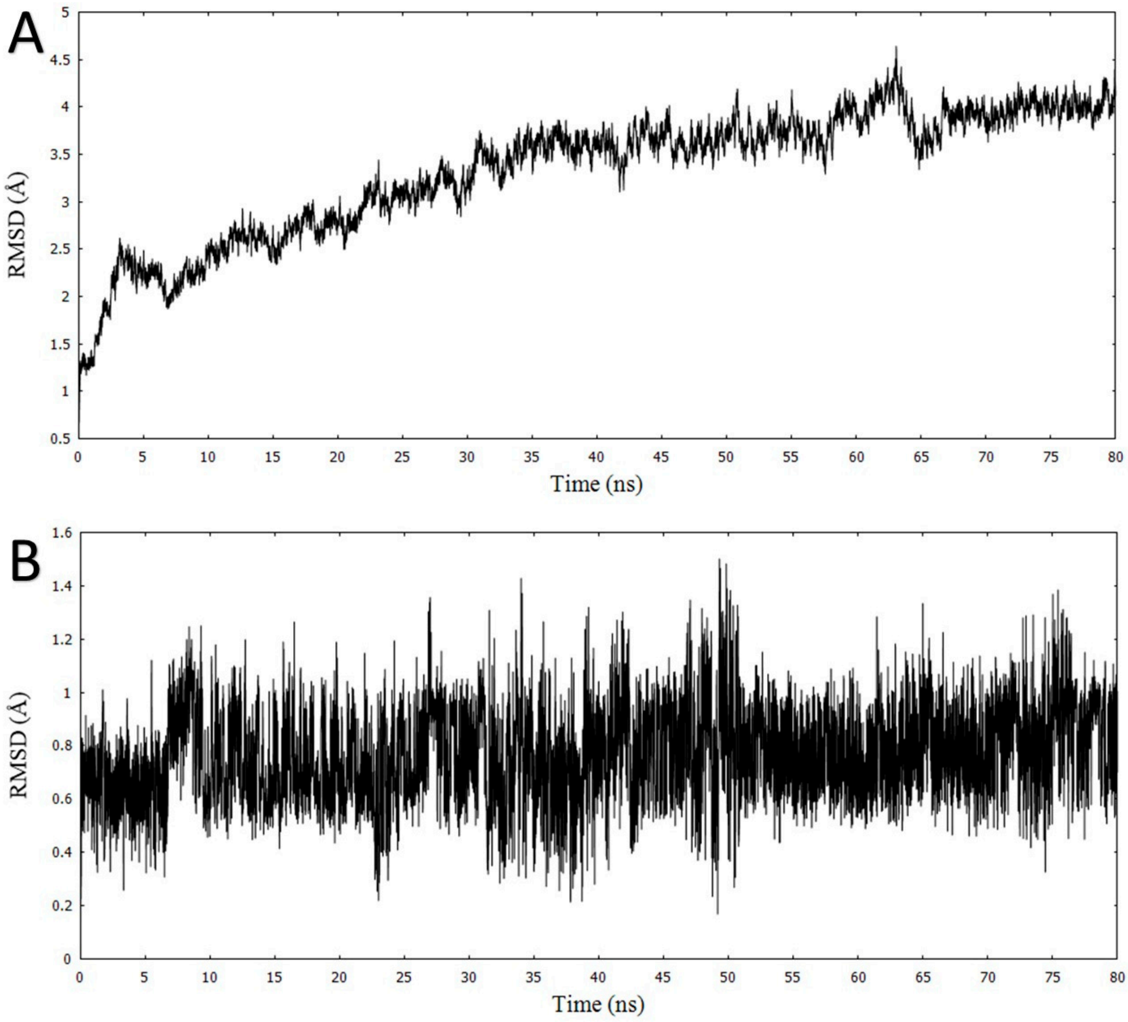

Figure S38. RMSD (Å) of the receptor (A) and the ligand (B) in the Cu T1 site complex analyzed over 80 ns.

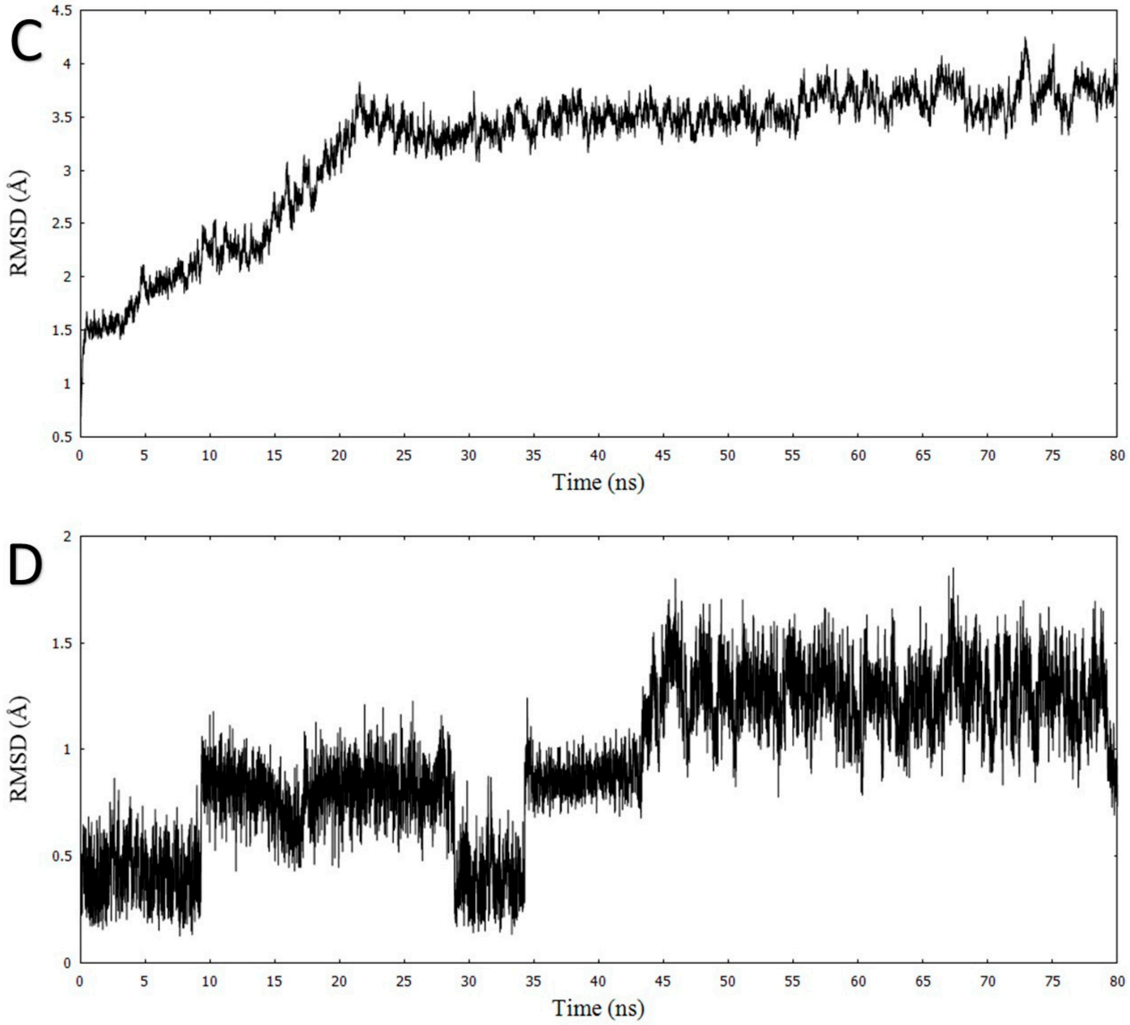

Figure S39. RMSD (Å) of the receptor (C) and the ligand (D) in the TNC site complex analyzed over 80 ns.

5.- Table H-bonds molecular dynamics

Table S1. Summary of hydrogen-bond interactions detected over 80 ns of molecular dynamics simulation between acetylated derivative of medicarpin (ligand) and the laccase (1GYC) receptor at the CuT1 (1) and TNC (2) binding sites.

(1) Site CuT1			(2) Site TNC		
Donor	Acceptor	Occupancy	Donor	Acceptor	Occupancy
ASN264	LIG	124.24%	LEU112	LIG	10.34%
HIS458	LIG	57.52%	LEU112	LIG	186.36%
PHE265	LIG	145.96%	TYR491	LIG	239.99%
GLY392	LIG	7.36%	LEU459	LIG	31.39%
GLY392	LIG	90.66%	PHE450	LIG	9.71%
PHE265	LIG	28.85%	SER113	LIG	71.14%
PHE162	LIG	68.92%	SER113	LIG	24.36%
PRO391	LIG	6.90%	LEU112	LIG	6.64%
LIG	ASP206	36.00%	PHE81	LIG	38.33%
LIG	HIS458	25.54%	PRO346	LIG	33.34%
LIG	ALA393	31.26%	LIG	LEU112	60.64%
LIG	GLY392	27.36%	LIG	TYR491	366.77%
LIG	PHE265	101.70%	LIG	SER113	9.04%
LIG	PRO163	29.81%	LIG	GLU460	32.29%
LIG	PHE162	93.63%	LIG	SER113	10.32%
LIG	PHE337	5.38%	LIG	PRO346	13.88%
ILE455	LIG	45.81%	LIG	GLN499	338.82%
ASP206	LIG	29.68%	PHE81	LIG	105.80%
LIG	ASN264	33.50%	LIG	LEU459	17.30%
PRO394	LIG	248.70%	LIG	PHE81	147.63%
ILE455	LIG	176.90%	GLU460	LIG	9.54%
LIG	ILE455	44.99%	ARG157	LIG	28.65%
ASN208	LIG	86.68%	LIG	GLU460	6.36%
LIG	PRO394	82.70%	LIG	ARG157	13.40%
ASN264	LIG	63.91%	LEU58	LIG	175.05%
LIG	ASP206	41.08%	LIG	LEU58	59.08%
LIG	ASN208	50.16%	LEU58	LIG	6.38%
LEU164	LIG	25.11%	GLN499	LIG	169.44%
LEU164	LIG	147.13%	ALA80	LIG	142.59%
LIG	LEU164	77.54%	LIG	ALA80	39.45%
LIG	LEU164	21.66%	ALA80	LIG	177.28%
LIG	PRO394	6.11%	ALA80	LIG	6.75%
PHE239	LIG	93.31%	LIG	GLU496	34.69%
LIG	PHE239	78.31%	GLU496	LIG	17.64%

LIG	PHE162	12.61%	SER60	LIG	242.49%
PRO431	LIG	49.01%	LIG	SER60	199.61%
ASN208	LIG	9.24%	PRO86	LIG	183.19%
PHE457	LIG	69.38%	LIG	PRO86	138.43%
LIG	PHE457	52.04%	GLU460	LIG	17.60%
LIG	GLN237	8.38%	GLN499	LIG	5.01%
SER427	LIG	49.09%	LIG	GLN499	5.79%
LIG	SER427	27.27%	PRO86	LIG	175.59%
PRO394	LIG	48.26%	LEU494	LIG	334.05%
THR430	LIG	113.79%	LIG	LEU494	70.28%
LIG	THR430	37.01%	LIG	LEU494	111.25%
LIG	PRO431	14.01%	LEU494	LIG	4.65%
LIG	THR428	32.20%	LIG	TYR491	86.63%
THR430	LIG	67.15%	LIG	GLY493	4.10%
GLY429	LIG	49.42%	LIG	CYS85	123.59%
ALA433	LIG	38.81%	GLN499	LIG	24.11%
LIG	ALA433	11.79%	GLU496	LIG	24.99%
LIG	SER427	5.79%	SER495	LIG	27.34%
ALA432	LIG	7.67%	LIG	GLU496	34.48%
ALA432	LIG	7.22%	LIG	SER495	70.05%
THR430	LIG	19.52%	CYS85	LIG	28.64%
ILE301	LIG	35.60%	LIG	ASP492	4.54%
LIG	ILE301	7.50%	CYS85	LIG	16.77%
LIG	THR430	27.43%	LIG	PRO86	8.90%
LIG	GLY429	26.27%	LIG	ASN498	5.08%
ALA410	LIG	41.51%			
LIG	ALA410	8.28%			
LIG	GLU302	18.21%			
THR430	LIG	7.45%			
THR303	LIG	45.88%			
LIG	THR303	19.05%			
LIG	ALA410	23.50%			
LIG	GLY411	31.07%			
GLY411	LIG	113.21%			
LIG	THR303	6.46%			
THR303	LIG	7.39%			
LIG	ALA433	5.64%			
LIG	GLY434	5.71%			
ASP435	LIG	6.19%			
ILE301	LIG	5.35%			
ASN304	LIG	9.28%			

LIG	ASN304	15.66%			
LIG	PRO299	14.79%			
PRO299	LIG	18.04%			
LIG	PRO299	5.58%			
GLN237	LIG	13.10%			
LIG	VAL297	9.11%			
LEU231	LIG	55.98%			
LIG	LEU231	30.96%			
LEU231	LIG	48.99%			
ILE298	LIG	124.92%			
LIG	ILE298	36.89%			
ILE298	LIG	5.47%			
LIG	ILE298	8.40%			
LIG	LEU232	82.38%			
LEU232	LIG	162.34%			
LIG	LEU232	37.20%			
LEU231	LIG	19.85%			
LEU232	LIG	54.14%			
LIG	PRO230	32.67%			
LEU232	LIG	37.69%			
LEU231	LIG	19.45%			
LEU232	LIG	39.66%			
LIG	LEU231	16.90%			
PRO230	LIG	30.86%			
LIG	PRO230	12.15%			
VAL233	LIG	10.80%			
ARG136	LIG	8.85%			
THR219	LIG	37.75%			
LIG	THR219	15.14%			
ASN251	LIG	5.99%			
ASN217	LIG	10.68%			
LIG	ASN217	5.81%			
GLN252	LIG	6.06%			
LIG	GLN252	6.22%			

6. Docking results of derivatives

Table S2. Binding energy (Kcal/mol), interaction frequencies (%) and ligand-amino acid residue interactions of medicarpin (**1**) and derivates (**2**-**12**) with the T1 and TNC sites of the enzyme (1GYC) by docking molecular.

Compounds	Binding energy (Kcal/mol)		Interaction frequency (%) *		Ligand-amino acid residue interactions
	T1	TNC	T1	TNC	
**1**	-7.57	NI	35	NI	His458 (H-bond), Pro391 (H-bond), Ile455, Asp206, Phe265, Phe162, Asn208, Gly392.
**2**	-7.51	NI	15	NI	His458 (H-bond), Ile455, Asp206, Asn264, Phe265, Pro391, Phe332.
**3**	-7.95	NI	15	NI	His458, Ile455, Pro394, Pro391, Phe332, Phe265.
**4**	-8.85	NI	5	NI	His458, Pro394, Ile455, Phe162, Phe332, Pro391, Phe265, Asp206.
**5**	-8.50	-7.63	33	9	His458 (H-bond), Asn264 (H-bond), Asn208 (H-bond), Phe265, Ile455, Pro391, Phe332, Pro207. Leu112, His111, Pro346, Ser110, Leu459, Glu460, Leu58.
**6**	-9.47	-8.57	10	2	Asn264 (H-bond), Asn208 (H-bond), His458, Phe332, Pro391, Ile455, Phe265, Phe239, Pro394. Leu112, His452, Tyr116, His109, His11, Pro346, Cu1501.
**7**	-9.12	-7.73	11	3	Asn264 (H-bond), Asn208 (H-bond), His458, Phe332, Pro391, Ile455, Phe265, Phe239, Pro394, Pro396. His111, Leu459, Phe81, Leu58, Tyr491 (H-bond), Pro346.
**8**	-7.55	-7.85	11	2	Gly392 (H-bond), Ala393 (H-bond), Ile455, His458, Phe162, Pro163, Phe457, Phe265, Pro431, Asp206. His111, Leu459, Pro346, Leu58, Ser113.
**9**	-7-48	-7.76	10	2	His458 (H-bond), Gly392 (H-bond), Ala393 (H-bond), Phe162, Pro163, Asn264, Phe457, Phe265, Pro431, Asp206, Ala388. His111, Leu459, Pro246, Leu112, Ser110, Ser113, Leu58.
**10**	-8.43	NI	15	NI	His458 (H-bond), Asp206, Phe162, Phe265, Ile455, Pro394, Asn264, Pro391.
**11**	-8.95	-8.83	12	1	His458, Gly392, Phe162, Phe332, Pro391, Pro163, Asn264, Ile455, Asp206, Phe239, Phe265, Pro394, Pro396, Ala393. His111 (H-bond), Leu459, Ser113, Pro346, Phe81, Pro347.

**12**	-835	NI	10	NI	His458 (H-bond), Ala393, Phe265, Phe162, Ile455, Asp206.

NI= No interaction detected

H-bond= Hydrogen bond interactions.

* Interaction frequency corresponds to the percentage of docking poses showing interactions at the indicated site.
